# Transcriptome profiling of banana shrimp (*Fenneropenaeus merguiensis*) ovaries and testes: Insights into *FoxL2*

**DOI:** 10.1371/journal.pone.0292782

**Published:** 2023-10-12

**Authors:** Wutthipat Potiyanadech, Chaturawit Choomee, Wilaiwan Chotigeat

**Affiliations:** Biological Science Division, Molecular Biology and Bioinformatics Program, Faculty of Science, Prince of Songkla University, Hat Yai, Songkhla, Thailand; Shanghai Ocean University, CHINA

## Abstract

The banana shrimp is found in the Pacific and Indian Oceans. Female shrimp are preferred for consumption because they are larger than males. Understanding the mechanism of sex differentiation is important for developing techniques to increase the number of female shrimp for economic benefits. This study investigates the reproductive development of *F*. *merguiensis* using transcriptome analysis. *Sxl2*, *dsx*, *AGH*, *FEM-1*, and *Nrg-X2* were classified as essential genes for testes development during the juvenile stage. Several genes were required for both juvenile and adult male development. Additionally, the expression of several genes was shown to be required for juvenile and adult ovarian development, including *SOP1*, *SOP2*, *Ptgr1*, *EST*, *Vgr*, *Vmol1*, and *TR-beta A*. Interestingly, high levels of *FoxL2* expression were observed in the testes, in contrast to previous studies in humans and other mammals. The binding of FoxL2 to the *Vtg* promoter was demonstrated *in silico* with the highest relative binding score (RS = 0.89) using the JASPAR program. Knock-down of the *FoxL2* gene with dsRNA significantly suppressed *FoxL2* at 2, 4, and 6 d. As a result, *Vtg* expression increased when compared with the control at 2, 4, and 6 d, indicating that *FoxL2* plays an important role in *Vtg* expression in the ovary. Our findings highlight the role of *FoxL2* in banana shrimp reproduction and provide valuable information on the genes associated with the *F*. *merguiensis* reproductive system.

## Introduction

The banana shrimp (*Fenneropenaeus merguiensis*) is a marine crustacean belonging to the family *Penaeidae*. It is native to the Pacific and Indian Oceans. Female shrimp are preferred over males for commercial purposes because they grow faster and have a larger body size than males [1, 2]. Therefore, the production of female shrimp is economically important. Understanding the mechanisms underlying sex differentiation in banana shrimp is essential for developing technologies to increase the abundance of female shrimp on the market [3].

Transcriptome analysis is a molecular biology technique used to study gene expression levels in biological samples. It has several benefits; it assists in identifying differentially expressed genes [4], understanding biological processes [5], the functional annotation of genes [6], developing diagnostic tools [7], and identifying alternative splicing events [8]. Recently, transcriptome analysis has been utilized to study the differences in gene expression levels in the reproductive systems of various shrimp species, such as brine shrimp (*Artemia franciscana*) [9], banana shrimp (*F*. *merguiensis*) [10], Pacific white shrimp (*Litopenaeus vannamei)* [6], Japanese mantis shrimp (*Oratosquilla oratoria*) [11], and littoral shrimp (*Palaemon serratus*) [4]. Forkhead box protein L2 (*FoxL2*) is a transcription factor that is involved in sex determination in female vertebrates [12, 13]. *FoxL2* is preferentially expressed in the ovary and plays a crucial role in ovarian differentiation and maintenance by repressing testis-specific genes [14].

The *FoxL2* gene plays a role in regulating the proliferation and differentiation of cells during ovarian development in humans [12, 13]. In mammals, *FoxL2* plays a vital role in the activation of the follicle-stimulating hormone (*FSH*) and is expressed in the ovaries, eyelids, pituitary gland, and follicular (granulosa) cells [12, 15–19]. *FoxL2* deficiency results in an increase in the expression of the SRY-box transcription factor 9 (*Sox-9*), doublesex and mab-3-related transcription factor 1 (*Dmrt1*) genes. In contrast, the expression levels of the wingless-type MMTV integration site family members 4 (*Wnt4*) and R-spondin 1 (*R-Spo1*) decreased in mice as a result of *FoxL2* deficiency [19–21]. Although these mice possess a female genotype, *FoxL2* deficiency leads to the development of a male organism [19–21]. In mammals and fish, *FoxL2* plays a role in activating the cytochrome P450, family 19, subfamily A, member 1 (*CYP19A1*), which participates in the synthesis of estrogen and aromatase [22–26]. The expression of male differentiation genes, including steroidogenic factor-1 (*Sf1*), gonadal soma-derived factor (*Gsdf*), and *Dmrt1*, increased after *FoxL2* and *CYP19A1* were knocked down. This led to male differentiation [27].

In the olive flounder (*Paralichthys olivaceus*), the *FoxL2* gene has been reported to control the expression of the *CYP19A* gene in collaboration with the nuclear receptor, subfamily 5, group A, member 2 (*Nr5a2*). This collaboration leads to the suppression of *Dmrt1*. However, increasing *FoxL2* expression in the male olive flounder did not reverse its sex to female. This finding indicates that increased expression of *FoxL2* alone is insufficient for inducing sex reversal [28]. Three homeologs of *FoxL2*: *Cgfoxl2a-B*, *Cgfoxl2b-A*, and *Cgfoxl2b-B*, have been reported in gibel carp (*Carassius gibelio*). *Cgfoxl2a-B* deficiency results in the arrest of complete sex reversal or ovarian development. Furthermore, the complete disruption of *Cgfoxl2b-A* or *Cgfoxl2b-B* resulted in germ cell depletion [29].

In crustaceans, *FoxL2* of the Pacific white shrimp was highly expressed in the testis [6], but no significant expression was observed in the ovary samples by transcriptome analysis [10]. These findings indicate variations in the expression of *FoxL2* between vertebrates and shrimp. *FoxL2* knock-down using RNAi in *Scylla paramamosain* increased vitellogenin (*Vtg*) expression in the ovary. In addition, the overexpression of *FoxL2* in *Eriocheir sinensis* resulted in a reduction of *Vtg* expression [30]. *FoxL2* binds DEAD (Asp-Glu-Ala-Asp) box RNA helicase 20 (*DDX20*) along with the fushi tarazu factor 1 (*Ftz-F1*), which is likely the mechanism behind the observed reduction in *Vtg* expression levels [30].

In this study, we aimed to explore the differential expression levels of genes in the reproductive system. A transcriptome analysis was conducted on testes and ovary specimens of juvenile and adult banana shrimp to identify genes involved in shrimp reproduction. The results of this analysis were further validated using quantitative real-time PCR (qPCR) on selected genes of interest. High levels of *FoxL2* transcripts were detected in the juvenile and adult testes of banana shrimp. This is in contrast to humans and other mammals, where *FoxL2* is highly expressed in the ovary [12, 15–19]. Therefore, the role of *FoxL2* in banana shrimp was also investigated.

## Materials & methods

### Sample collection

A juvenile *F*. *merguiensis* was obtained 3 days post-larvae (PL3) that was produced using a wild-caught broodstock in a farm in the Nakhon Si Thammarat province of Thailand. The PL3 shrimp were reared in seawater with a salinity of 30 parts per thousand (ppt) at 28–30 °C in a concrete pond at the aquaculture building, Faculty of Science, Prince of Songkla University. They were fed *Artemia* four times a day until ~PL10. From PL10 to PL15, they were fed a crushed commercial shrimp feed mixed with *Artemia* four times a day. From PL15 to two months post-larvae, they were fed *Artemia* mixed with shrimp pellets three times daily, and thereafter, they were fed with shrimp pellets. When the shrimp appeared an external sex organ, the shrimp’s length was measured and used as the juvenile shrimp. The adults *F*. *merguiensis* were wild-caught (13–14 cm body length, 14–20 g weight) shrimp before they became broodstock shrimp. They were obtained from the same farm mentioned above and were reared in a concrete tank at the aquaculture building as described above. These shrimps used for the transcriptome experiment were sacrificed by knocking in the ice-cold water for 5–10 min and decapitation, then separated the ovaries and testes for the investigation.

The shrimp used for the *Foxl2* dsRNA experiment was the wild-caught adult shrimp with the length of a proximate size (15.61 ± 1.04 cm) and weight (28.48 ± 5.59 g) of the broodstock shrimp. The shrimp were reared for 2 wk to achieve the undeveloped ovary before the experiment. The shrimp in this experiment were anesthetized by placing them on ice for 30 sec. Then the shrimp was quickly injected with the *Foxl2* dsRNA and softly put the shrimp back into the water tank. The *Foxl2* dsRNA post-injection shrimps for 2, 4, and 6 d were sacrificed as described above for separating the ovaries and testes for the investigation.

### Animal ethics statement

All animal experimental procedures were performed under the relevant guidelines and regulations and were approved by the Institutional Animal Care and Use Committee, Prince of Songkla University.

### Animal selection for RNA sequencing

**Fig 1:**
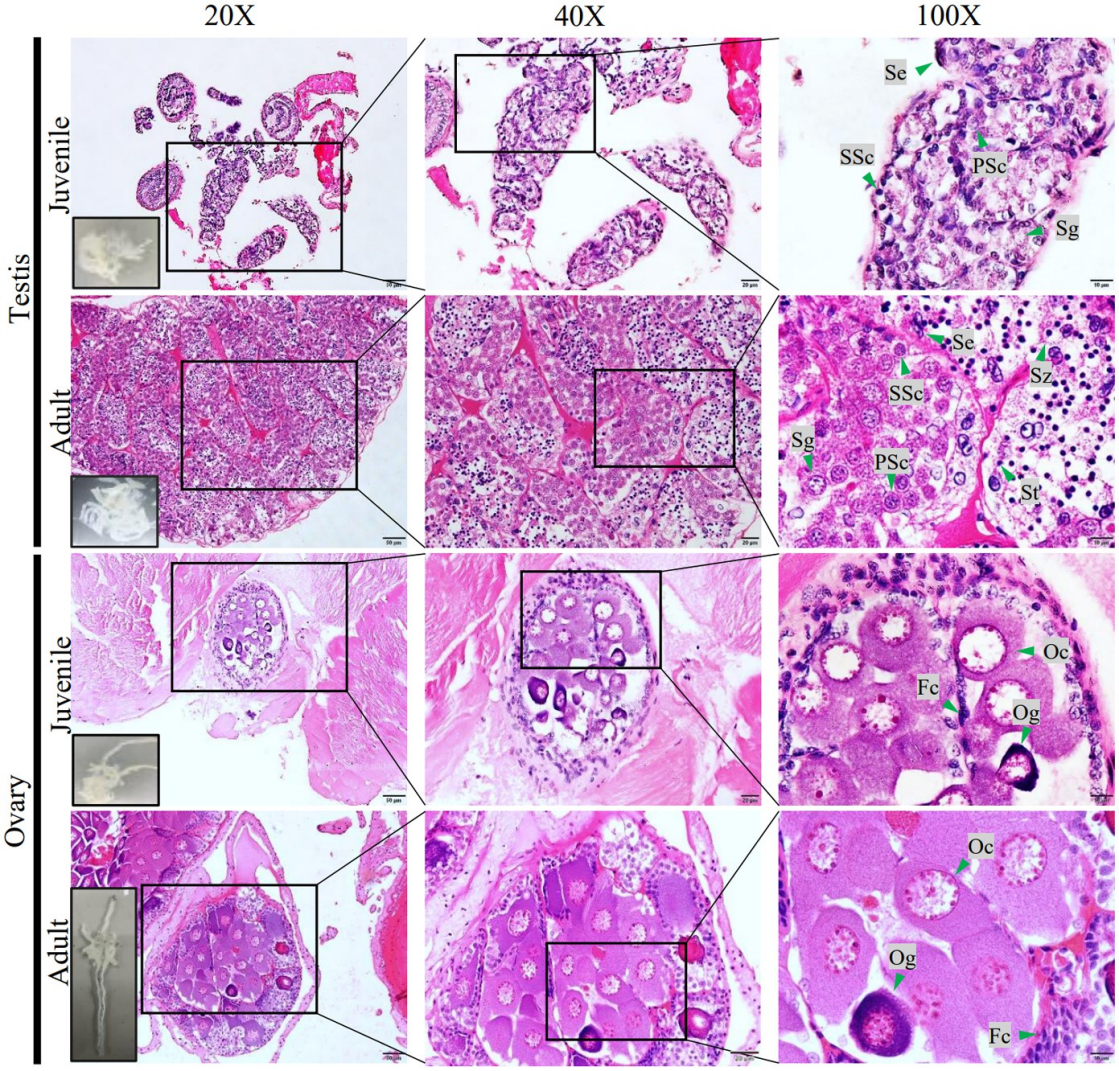
H&E staining section of the testis and ovarian samples used for transcriptome analysis. Transverse section (5μm thick) of the testis and ovarian samples at the same stage as the sample used for transcriptome sequencing. Sg: spermatogonial stem cells; PSc: primary Spermatocyte; SSc: secondary Spermatocyte St: spermatids; Sz: spermatozoa; Se: Sertoli cell; Og: Oogonia; Oc: oocytes; Fc: follicular cells.

The shrimp were randomly killed by soaking in ice for 5 min. The ovary and testis sections were performed H&E staining for histological screening, according to Chimnual and colleagues [31]. Shrimp were defined as juveniles at the first appearance of external gonads and when their body length measured 7–8 cm (3.0–3.8 g) for females containing ovaries with abundant oogonia and few oocytes. The males were 6–7 cm long (2.5–3.6 g), having testes with many spermatogonia and without spermatid cells (Fig 1).

The adults *F*. *merguiensis* (13–14 cm body length, 14–20 g weight) were randomly prepared for histological screening as described above. The adult samples were testes containing somatic stem cells, spermatogonia, spermatocyte, spermatid cells, and a more pronounced loop division. The ovary of adult banana shrimp contained oogonia, oocytes, and previtellogenic oocytes < 65 μm in size. The nucleus of each oocyte is composed of prominent granular nucleoli (Fig 1). The three shrimp were sacrificed, dissected for the ovaries and testes, and pooled for RNA extraction and sequencing.

### RNA preparation

Total RNA was extracted from the pooled sample (three testes and three ovaries from juvenile and mature shrimp) using TRIzol^®^ reagent (Thermo Fisher Scientific Inc., CA, USA) according to the manufacturer’s protocol. Briefly, the shrimp tissue (50–100 mg) was homogenized in TRIzol^®^ reagent (1 mL), chloroform (0.2 mL) was added, and it was incubated for 3 min. The sample was spun at 12,000 x g at 4 °C for 15 min, and the aqueous phase was transferred into a new tube. Isopropanol (0.5 mL) was added to the aqueous phase and spun at 12,000 x g at 4 °C for 10 min. The pellet was washed with cold 75% ethanol (1 mL) and spun at 7,500 x g at 4 °C for 5 min. The RNA pellet was air-dried and suspended in 50 μL RNase-free water. After the total RNA was determined, RNA quality and quantity were analyzed using a 2100 Bioanalyser (Agilent, CA, US).

### RNA sequencing and data processing

RNA sequencing and data processing were carried out using BGI (BGI, Shenzhen, China). The BGISEQ-500 transcriptome library construction protocol (BGI, Shenzhen, China) was used to construct a transcriptome library from total RNA. Briefly, mRNA fragments were converted into double-stranded cDNA (dscDNA) by reverse transcription using the N6 random primer. The dscDNA was then subjected to end-repair and 3′ adenylation. Adaptors were then ligated to the 3′ adenylated ends of the cDNA fragments. The purified products were used to perform PCR amplification to increase the number of cDNA templates. The PCR product was heat-denatured to convert dsDNA to ssDNA. The ssDNA was cyclized using splint oligo and DNA ligase. Sequencing was performed using the BGISEQ-500 platform (BGI, Shenzhen, China).

Raw reads were then filtered, and de-novo assembly, functional annotation, and differentially expressed gene (DEG) detection were performed using BGI. Briefly, adaptor sequences were removed from the reads; and reads with more than 5% unknown bases (N) and low-quality reads (reads were considered low-quality if 20% of that read, or more, scored less than 15 for quality) were removed from the transcript data to obtain clean reads. De-novo assembly was performed on the clean reads using Trinity (version: 2.06), and transcripts were grouped into unigene clusters using TGICL (version: 2.06). For gene annotation, BLAST (version: 2.2.23) and Diamond (version: 0.8.31) software were used to align the unigenes to the non-redundant nucleotide, non-redundant protein, KOG, KEGG, and SwissProt databases. Software Blast2GO (version: 2.5.0) was used for GO annotation. InterProScan5 (version: 5.11–51.0) was used for InterPro annotation. PossionDis software was used to detect DEGs between the testes and ovaries samples. When comparing the ovaries and testes of juveniles and adults, genes were considered significantly differentially expressed if the false discovery rate *p-value* (FDR *p-value*) ≤ 0.001 and the absolute value of a log_2_fold-change (ovary expression/testis expression) was ≥ 1.

### Validation of DEGs of the transcript data by quantitative real-time PCR analysis

Quantitative real-time PCR was performed to validate the RNA sequencing gene expression data. To analyze gene expression, we selected four unigenes that are involved in sex differentiation and sex development. The primers for doublesex (*dsx*), *FoxL2*, *Vtg*, and ovarian peritrophin 1 (*SOP1*) were designed (Table 1). Three technical sample replicates were used to quantify gene expression from the ovaries and testes of *F*. *merguiensis* in the juvenile and adult stages. RNA was converted to cDNA in a total reaction volume of 20 μL. This included 1 μg of RNA (1 μL) and 100 ng of the random primer (1 μL). Samples were incubated at 70 °C for 5 min and then placed on ice for 5 min. The 5x reverse transcription buffer (4 μL), 4 U/μL AMV (0.5 μL), and 10 mM dNTP (1.25 μL) were added to the reaction; distilled water was added to reach 20 μL, and samples were incubated at 37 °C for 1 hr. The qPCR reaction of 12.5 μL contained 700 ng of cDNA (1 μL), 0.4 μL of 10 μM of the forward and reverse primer, 2X FastStart Universal SYBR Green Master (6.25 μL) [Roche, Mannheim, Germany], and distilled water was added to reach 12.5 μL. PCR was performed using the Mx3000PTM (Stratagene, CA, USA) under the following conditions: initial denaturation at 95 °C for 5 min, 40 cycles of amplification [94 °C for 30 sec, (specific annealing temperature for each primer, Table 1) for 30 sec, 72 °C for 45 sec]. *β*- actin was used as an internal control. The relative expression level of the selected unigene was calculated based on the 2^−ΔΔCT^ method [32].

**Table 1:** Primer sequences for quantitative real-time PCR analysis used to verify RNA sequencing gene expression data.

Primer name	Primer sequences (5′→3′)	Annealing temperature
F-*dsx*	AACGCTGAGGGAGTTTGTTG	61 °C
R-dsx	CCTGAAGTTGTTGCTGTTGC	
F-*FoxL2*	GCTACAGCTTAGCGAAATCT	54 °C
R-*FoxL2*	GTCTTCGTGGTTGGGGTCTA	
F-*FoxL2* Full length	CCGGATCCATGACTTCCCTGGA	60 °C
R-*FoxL2* Full length	GGGTCGACCTATATTTTCGAATC	
F-*FEM1*	CCCCATTTGTACTTTCCCAAGC	60 °C
R-*FEM1*	TGCTAGGCACTGTAAGGAAGTG	
F-*Tra-2c*	TACACAACCGAGAGACAGCTTC	60 °C
R-*Tra-2c*	ACCCATGTATATGCCAGGAGTG	
F-*Sxl2*	TCATCAACTACCTGCCACAGAC	60 °C
R-*Sxl2*	ACCTTGATGCGTTTGTGCTG	
F-*SOP1*	TTATGTTGTGTTGGCCCTGG	55 °C
R-*SOP1*	ACCAAGGTCTTGCAGTTGGC	
F-*Vtg*	ATCTCACCTGGATCAGCCCT	57 °C
R-*Vtg*	GAGGACTCGGAGATGAAGCG	
F-*β-actin*	CAGATCATGTTTGAGACCTTC	55 °C
R-*β-actin*	GATGTCCACGTCCACTTCAT	

### Sequence and phylogenetic analysis of *FmFoxL2*

*FmFoxL2* cDNA sequence (GenBank OQ870552) was derived from the transcriptomic data. The FmFoxL2 protein sequence was translated *FmFoxL2* cDNA sequence using bioinformatics tools. Nucleotide sequence similarity was analyzed using the NCBI BLAST tool (http://blast.ncbi.nlm.nih.gov/Blast.cgi). Multiple sequence alignments were performed using Clustal Omega (https://www.ebi.ac.uk/Tools/msa/clustalo/), and a phylogenetic tree was constructed using the neighbor-joining method in MEGA 11.0 (http://www.megasoftware.net/), with bootstrap values calculated from 1000 replicates.

Functional domains of the protein were predicted using the ExPASy PROSITE tool (https://prosite.expasy.org/). *FoxL2* domains were predicted using SMART (http://smart.embl-heidelberg.de/). The molecular size and theoretical isoelectric point of the protein were determined using the ExPASy Compute pI/Mw tool (https://web.expasy.org/compute_pi/). Signal peptides in the protein sequence were predicted using the SignalP 6.0 software (https://services.healthtech.dtu.dk/service.php?SignalP-6.0). N-glycosylation and phosphorylation sites were indicated using the NetNglyc 1.0 and NetPhos 3.1 servers (https://services.healthtech.dtu.dk), respectively.

### Prediction of the FmFoxL2 protein *Vtg* promoter binding site

A three-dimensional structure of the FoxL2 protein was constructed using the SWISS-MODEL software (https://swissmodel.expasy.org/interactive). Human *FOXL2* with a PDB ID of 7vou.1.C was used as a template. The *Vtg* binding site of the FoxL2 protein was analyzed using the JASPAR online software (http://jaspar.genereg.net/). According to no promotor part of *FmVtg* and no closely related shrimp *Vtg* promotor part were available on the NCBI database. Therefore, the mud crab *Vtg* promotor part was used to predict the banana shrimp FoxL2 *Vtg* promoter binding site, as reported previously [33].

### Knock-down of *FoxL2 in vivo* by double-stranded RNA

The *FoxL2* gene was knocked down to study the role of *FoxL2* in the ovarian development of female banana shrimp, based on a report that focused on *S*. *paramamosain* [33]. *FoxL2* of *F*. *merguiensis* was designed from position 600–801, which is in the *FoxL2* domain. The *FoxL2* fragment was amplified using the forward and reverse primers specified for *FoxL2* in Table 1, cloned into pGEM^®^-T Easy, and sequenced to verify the *FoxL2* fragment. dsRNA-*FoxL2* was synthesized using the T7 RiboMAX^™^ Expression RNAi System kit (Promega, WI, USA). The integrity of dsRNA was analyzed by 1.2% agarose gel electrophoresis. The concentration of dsRNA was quantified using a NanoDrop 2000.

The dsRNA was diluted to 0.75 μg/μL with phosphate buffer saline (PBS), and 3 μg of dsRNA per gram of shrimp was injected into the shrimp as in previous studies [34, 35]. Female shrimp measuring 15 ± 0.5 cm in length and 26 ± 2.8 g in weight were used in this experiment. One group was injected with ds*FoxL2* (three shrimp per group), and the other was injected with PBS (the negative control group). Repeat injections of *FoxL2* dsRNA were administered 2, 4, and 6 d after the initial injection. After injection at 2, 4 d, and 6 d, the ovaries of three shrimp from each group were collected as described above. Each ovary was divided into two parts (left lobe and right lobe). The first part of the ovary was inspected the ovarian development by the histological method as previously described in the section “Animals selection for RNA sequencing”. The other part of the ovary was used to analyze the expression levels of *Vtg* and *FoxL2*, genes using the same method described in the section “Confirmation of the transcript data by quantitative real-time PCR analysis”.

### Statistical analysis

Each experiment was performed in triplicate, and the results were presented as means ± standard deviations (SD). T-test was used to analyze the differences between the samples in each experiment. Differences were considered statistically significant if the *p-value* < 0.05. Statistical analyses were performed using GraphPad Prism software (version: 9.3.0).

## Results

### Quality of RNA sequencing and assembly analysis

**Fig 2:**
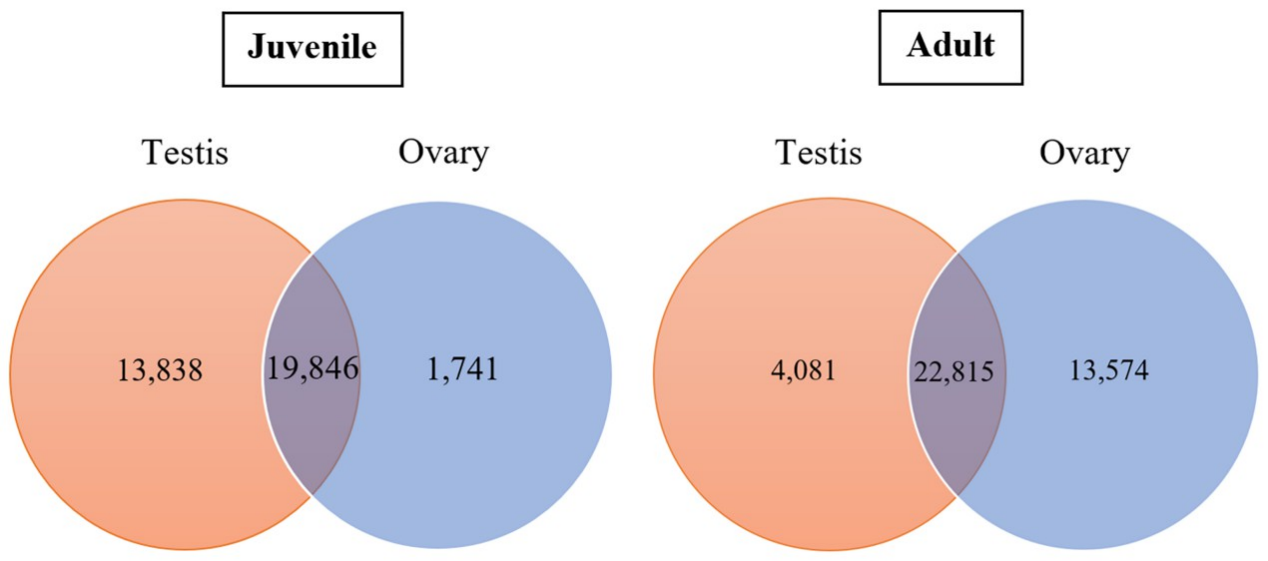
Circular diagrams showing DEGs number of testis and ovary samples. The DEGs of the testis and ovary samples from juvenile and adult banana shrimp are present in a circular chart. The overlap area was the repetitive DEGs between the testis and ovary of each stage.

Four cDNA libraries were constructed from the ovaries and testes of *F*. *merguiensis* at the juvenile and adult stages. The BGISEQ-500 sequencing platform generated reads for the juvenile testes and ovaries and the adult testes and ovaries at 77.13, 77.13, 71.87, and 73.62 million reads (MR), respectively (Table 2). After removing the adaptor sequences and filtering out low-quality and unknown base (N) reads, high-quality clean reads of the transcript data were achieved for the juvenile testes (69.25 MR) and ovary (70.39 MR) and the adult testes (66.31 MR) and ovaries (65.39 MR). The number of unigenes in the testes was greater than in the ovaries. The total number of DEGs was higher in the juvenile testes than in the adult testes. In contrast, the DEGs in the ovaries were more abundant in the adult ovaries than in the juvenile ovaries. In addition, the DEGs were approximately 19,846 and 22,815, overlapped between the testes and ovaries of the juvenile and adult shrimp, respectively (Fig 2). The unigenes’ mean length and N50 length were reasonable for both the testes and ovaries samples (Table 2). The RNA-seq data from this study was submitted to the NCBI database with the following project numbers: PRJNA961319 for testes and PRJNA997123 for ovaries, respectively.

**Table 2:** Summary of the generated transcript data of testes and ovaries from the *F*. *merguiensis* juvenile and adult stage. The values in the brackets^1^ and brackets^2^ represent the percentage of clean read and unigenes, respectively.

Samples	Juvenile		Adults	
	Testes	Ovaries	Testes	Ovaries
Total number of raw reads (MR)	77.13	77.13	71.87	73.62
Total number of clean reads (MR)	69.25(89.79%)^1^	70.39(91.26%)^1^	66.31(92.26%)^1^	65.39(88.82%)^1^
Total number of unigenes	38,216	24,503	77,809	43,211
The mean length of unigenes (bp)	1,159	979	725	769
N50 length of unigenes (bp)	2,295	1,786	1,426	1,511
Total number of DEGs (unigenes)	13,838(36.2%)^2^	1,741 (9.5%)^2^	4,081(5.2%)^2^	13,574 (31.4%)^2^

### Unigene annotation

**Fig 3:**
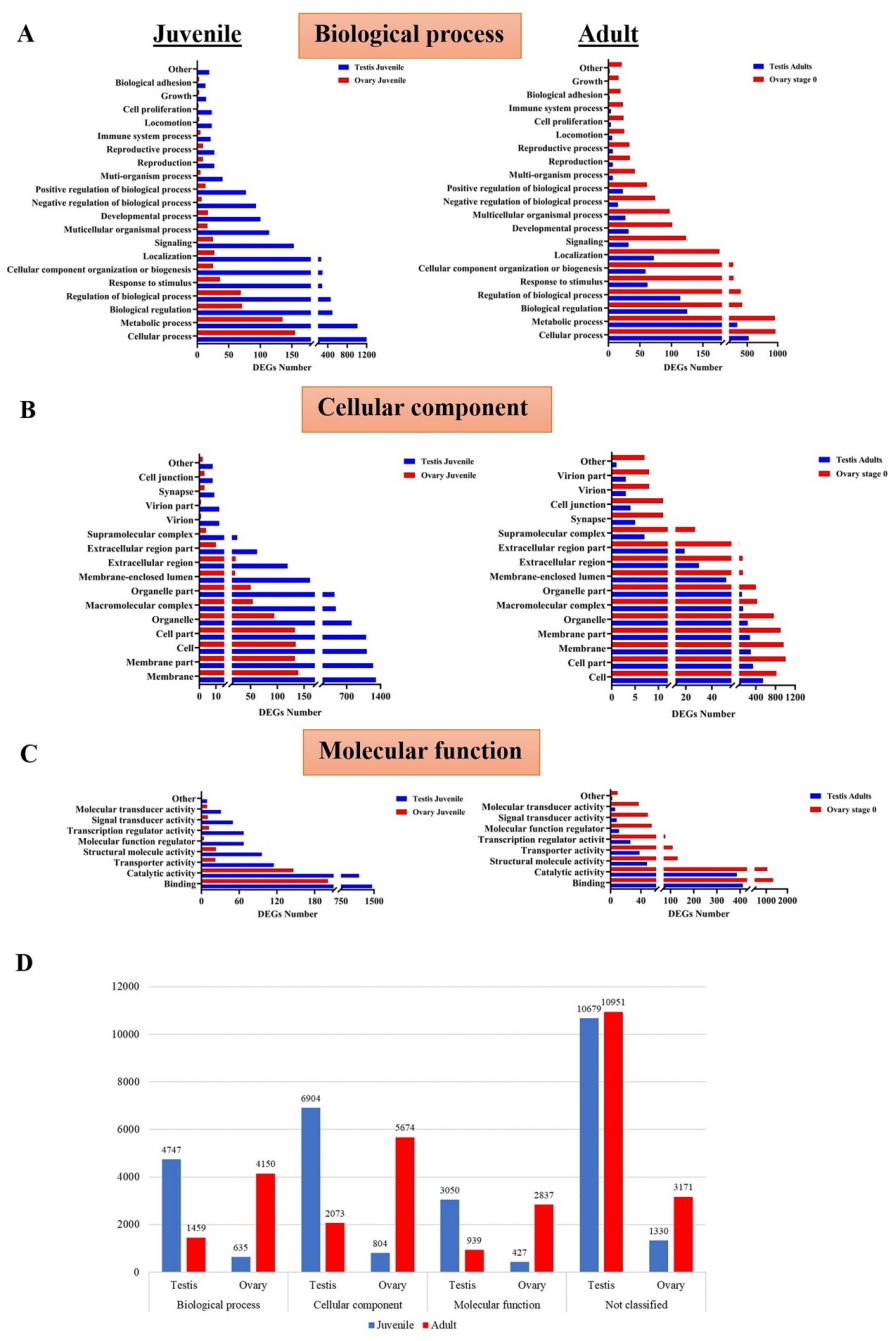
The DEGs between the testes and ovaries of *F*. *merguiensis* were classified using Gene Ontology (GO) terms. The GO terms are (A) biology process, (B) cellular component, and (C) molecular function. X-axis: The number of DEGs; Y-axis: Ontology sub-categories of genes. (D) Summary of the number of DEGs in each GO term. X-axis: DEGs in GO Terms, and Y-axis: The number of DEGs. The blue bars show the number of DEGs in the juvenile shrimp gonad. The red bars show the number of DEGs in the adult shrimp gonad.

The numeric depiction of unigenes in Table 2 and Fig 2 illustrates the number of unigenes obtained through the analysis of the PossionDis software. The testes and ovaries transcriptome data from the juvenile and adult stages were categorized into biological, cellular, and molecular processes using GO (Fig 3A–3C). A large number of genes were expressed in the biological processes and cellular processes of the testes and ovaries in both the juvenile and adult stages. Fewer genes were expressed in the molecular process compared to the other processes. The number of genes expressed in the juvenile stage for each GO term was significantly lower in the ovaries when compared to that of the testes. Conversely, the number of genes expressed for each GO term in the adult ovaries samples was higher than that in the testes (Fig 3D). It is important to clarify that a unigene is categorized when it associates with one or more interconnected GOs.

### Analysis of DEGs

In the juvenile stage, the number of DEGs in the testes (13,838 unigenes) was greater than that of the ovary (1,741 unigenes). In comparison, the number of DEGs in the adult testes (4,081 unigenes) was less than that of the ovaries (13,574 unigenes) (Table 2). When comparing DEGs in the testes and ovaries, unigenes with a false discovery rate *p-value* (FDR *p-value*) ≤ 0.001 and the absolute value of a log_2_fold-change (ovaries expression/testes expression) ≥ 1 were considered significantly differentially expressed. This suggests higher levels of gene expression during the development of the testes in the juvenile stage when compared to that of ovarian development. In contrast, more genes were expressed in the ovaries of adult shrimp when compared to those in the testes, likely because female adult shrimp develop a mature ovary. Therefore, this study classified DEGs into two groups: DEGs in the testes of the juvenile and adult shrimp and DEGs in the ovaries of the juvenile and adult shrimp.

### DEGs in the testis of the juvenile and adult shrimp

DEGs in the testis were separated into two groups. The first group was the DEGs for juvenile testes development, and five highly DEGs in this group included *dsx*, Sex-lethal 2 (*Sxl2*), androgenic gland hormone-like protein (*AGH*), Protein fem-1 homolog (*FEM-1*), and neuroglian-like isoform X2 (*Nrg-X2*) (Table 3). These DEGs were identified as essential for early testis development in the juvenile stage. This was demonstrated by the higher negative ratio observed in the transcript DEGs of the juvenile ovary/testis when compared to that of the adult stage. For example, the log_2_fold changes of the *dsx* and *Sxl2* transcripts for ovary/testis were -6.98 and -6.57 and increased to -2.50 and -1.93 in the adult stage, respectively. These genes are mainly involved in sex differentiation. *dsx* from *Fenneropenaeus chinensis* was reported to play a crucial role in male sexual differentiation in crustaceans [36]. In contrast, the *Sxl* gene is involved in sex determination and sexual differentiation in the female development of *Drosophila* sp. [37]. Another two genes are known as key regulators of male sexual differentiation, the *AGH* and *FEM-1* genes. *AGH* is the insulin-like androgenic gland hormone (*IAG*) and plays a crucial role in regulating male sexual differentiation in the Chinese shrimp, *F*. *chinensis* [38], and the giant freshwater shrimp, *Macrobrachium rosenbergii* [39]. The *FEM-1* gene has been shown to be specifically expressed in spermatogonia and was suggested to play a role in gamete formation in male gametes of both *Caenorhabditis elegans* and *L*. *vannamei* [40]. *Nrg-X2* is an isoform of the neuroglian (*Nrg*) gene family that involves forming septate junctions in various tissue of insects. During spermatogenesis, septate junctions are crucial permeability barriers at an early stage for appropriate sperm development. In addition, the knock-down of septate junctions disrupted the integrity of the permeability barrier, finally leading to sterility [41, 42].

**Table 3:** Differentially expressed genes (DEGs) in the testes of juvenile and adult banana shrimp.

Gene annotation	Log_2_fold-change		FDR *p-value*	
	Ovary/Testis of Juvenile	Ovary/Testis of Adults	Ovary /Testis of Juvenile	Ovary/Testis of Adults
**DEGs for juvenile testes development**				
Crustacean hyperglycemic hormone 1 (*CHH1*)	-11.24	NA	0.00	NA
SRY-box transcription factor 5 (*Sox5*)	-8.26	NA	4.35E-73	NA
Doublesex (*dsx*)	-6.98	-2.50	4.74E-41	4.63E-11
Sex-lethal 2 (*Sxl2*)	-6.57	-1.93	0.00	9.99E-144
Neuroglian-like isoform X2 (*Nrg-X2*)	-5.69	-3.82	0.00	0.00
Androgenic gland hormone-like protein (*AGH*)	-5.21	-1.63	1.82E-01	3.95E-01
Neuroglian-like isoform X1 (*Nrg-X1*)	-5.04	NA	2.42E-144	NA
Protein fem-1 homolog (*FEM-1*)	-4.92	-1.21	1.22E-195	1.18E-54
Retinoid X receptor 2	-3.17	0.37^a^^,^ ^b^	1.13E-05	8.08E-01
Transformer-2a (*Tra-2a*)	-2.96	-0.70	9.44E-113	1.66E-24
Kinesin-like protein KIFC1 (*Kifc1*)	-2.18	-0.70^a^	4.90E-147	1.90E-01
Beta-catenin (*β*-catenin)	-2.05	-0.76^b^	5.60E-288	8.98E-14
Nuclear progesterone receptor (*PGR*)	-1.79	-0.04^a^	0.00	1.50E-01
Male-specific lethal 3 (*Msl3*)	-1.61	0.13^a^^,^ ^b^	5.03E-29	1.61E-01
Cathepsin D (*Ctsd*)	-1.53	0.88^b^	4.35E-239	2.07E-217
**DEGs for both juvenile and adult testes development**				
Disrupted meiotic cDNA (*Dmc1*)	-8.51	-9.91	0.00	0.00
Heat shock protein 70 (*Hsp70*)	-7.84	-7.44	0.00	0.00
Hematopoietic prostaglandin D synthase (*Hpgds*)	-7.83	-7.47	1.61E-07	2.75E-10
Forkhead box L2 (*FoxL2*)	-7.52	-7.14	3.17E-50	0.00
Follistatin-related protein 5 (*Fst5*)	-6.44	-7.81	9.20E-165	3.00E-82
SRY-box transcription factor 14B (*Sox-14B*)	-5.73	-7.55	2.72E-60	1.08E-05
Lutropin-choriogonadotropic hormone receptor-like (*LHCGR*)	-5.13	-7.75	1.39E-09	4.69E-09
Farnesoic acid-O-methyl transferase (*FAOMeT*)	-4.58	-4.35	0.00	0.00
Juvenile hormone epoxide hydrolase (*JHEH*)	-4.11	-2.42	0.00	9.98E-242
Ecdysone receptor (*EcR*)	-4.09	-7.03	2.90E-08	2.84E-38
*Piwi-1*	-3.82	-3.90	7.28E-92	2.30E-101
Protein fem-1 homolog B (*FEM-1b*)	-3.74	-2.55	2.59E-09	1.75E-20
Transformer-2 (*Tra-2*)	-3.74	-3.52	2.15E-253	3.89E-17
Tudor and KH domain-containing protein (*TDRKH*)	-3.59	-3.43	8.42E-47	1.13E-165
*Vasa*	-3.09	-1.62	0.00	0.00
*Piwi-2*	-2.77	-2.02	0.00	0.00
Fushi tarazu-factor 1 (*Ftz-F1*)	-2.10	-2.10	2.22E-29	9.81E-43

NA indicates that no transcriptome data were found.

^a^ gene expression is not different between the testis and ovary because FDR > 0.001.

^b^ gene expression is not different between the testis and ovary because the absolute value of log_2_fold-change is less than 1.

The second group of genes showed high levels of differential expression in both the juvenile and adult testes (Table 3). These DEGs were required for both juvenile and adult testes development, for example, disrupted meiotic cDNA (*Dmc1*), hematopoietic prostaglandin D synthase (*Hpgds*), *FoxL2*, and follistatin-related protein 5 (*Fst5*). *Dmc1*, a DNA recombinase and RecA/Rad51 superfamily member plays a crucial role in meiotic recombination [43]. In the testis samples of *Penaeus monodon* [44] and *L*. *vannamei* [45], *Dmc1* expression was significantly upregulated. *Hpgds*, or *PGDS*, is an enzyme that catalyzes producing *PGD2* in the peripheral tissues [46]. *PGDS* is expressed in tissues such as the testis and ovaries [47, 48]. *PGDS* was highly expressed in the hepatopancreas, and the testis regulates spermatogenesis in the Chinese mitten crab *Eriocheir sinensis* [49]. *FoxL2* synthesized estrogen and aromatase in mammals and fish [22–26] and was involved in *Vtg* expression by binding to the *Vtg* promoter [33]. Therefore, low expression levels of *FoxL2* are directly related to the increase in *Vtg* expression observed in *E*. *sinensis* [30] and *S*. *paramamosain* [33]. Follistatin (*Fst*) is a vital regulatory protein of the transforming growth factor-beta (*TGF-β*) superfamily in vertebrates [50].

### DEGs in the ovary between juvenile and adult shrimp ovary

A total of nine genes were found to be highly differentially expressed in the juvenile and adult ovaries. These genes included ovarian peritrophin (*SOP*), *SOP1*, ovarian peritrophin 2 (*SOP2*), prostaglandin reductase 1 (*Ptgr1*), vitelline membrane outer layer protein 1 (*Vmo1*), estrogen sulfotransferase (*EST*), vitellogenin receptor (*Vgr*), thyroid hormone receptor beta-A (*TR-beta-A*), and transformer isoforms C (*Tra-2c*) (Table 4). Most of the genes in this group are involved in structural formation; *SOP* is a key component of the jelly layer and cortical rods in shrimp and plays a crucial role in protecting eggs during spawning [51]. *Ptgr1* is involved in polyunsaturated lipid proliferation during vitellogenesis in freshwater crayfish [52, 53]. Vmo1 is a protein that separates the yolk from the egg, is located in the outer layer of the egg white vitelline membrane, and protects the embryo against bacterial infection [54]. In addition, VmoI was reported to be produced in the hepatopancreas and transported into oocytes during the vitellogenesis of *L vannamei* [55]. Vgr is a plasma membrane-bound protein that specifically binds and mediates the transport of the protein Vtg into oocytes [56]. *Vtg*, Transformer-2 (*Tra-2*) was the only regulator gene in this group that was found to be differentially expressed. It has been reported to play a crucial role in sex differentiation and development in *Drosophila melanogaster* by directing the sex-specific alternative splicing of the *DSX* pre-mRNA in conjunction with the transformer (*Tra*) protein. Three isoforms, *Tra-2a*, *Tra-2b*, and *Tra-2c*, have been reported. *Tra-2c* has been shown to play a role in female determination in Chinese shrimp [3]. Other DEGs that were required during the adult stage included progestin membrane receptor component 1 (*Pgmrc1*), juvenile hormone esterase-like protein 1 (*JHE1*), and Chorion peroxidase-like (*Pxt*).

**Table 4:** Differentially expressed genes in the ovaries of juvenile and adult shrimp.

Gene annotation	Log_2_fold-change		FDR *p*-value	
	Ovary /Testis of Juvenile	Ovary/Testis of Adults	Ovary /Testis of Juvenile	Ovary/Testis of Adults
**DEGs for both juvenile and adult ovary development**				
Ovarian peritrophin 1 (*SOP1*)	20.53	15.39	0.00	0.00
Ovarian peritrophin 2 (*SOP2*)	19.64	15.09	0.00	0.00
Prostaglandin reductase 1 (*Ptgr1*)	18.05	12.02	0.00	0.00
Ovarian peritrophin (*SOP*)	14.40	8.50	0.00	0.00
Vitelline membrane outer layer protein 1 (*Vmo*1)	8.39	5.53	3.10E-107	8.05E-134
Transformer-2c (*Tra-2c*)	2.20	1.03	3.03E-09	4.85E-10
Estrogen sulfotransferase (*EST*)	10.35	12.56	3.24E-75	0.00
Vitellogenin receptor (*Vgr*)	8.62	13.67	0.00	0.00
Thyroid hormone receptor beta-A (*TR-beta-A*)	7.60	10.74	2.33E-17	2.93E-270
Vitellogenin (*Vtg*)	2.58^a^	10.24	1.06E-01	0.00
Juvenile hormone esterase-like protein 1 (*JHE*1)	-3.91^a^	6.63	9.63E-02	1.26E-12
Chorion peroxidase-like (*Pxt*)	-7.25	5.32	2.75E-09	1.03E-04
Wingless-type MMTV integration site family, member 4 (*Wnt4*)	NA	4.86	NA	9.41E-08
Octopamine receptor beta-2R (*Octbeta2R*)	NA	3.32	NA	1.24E-32
Argonaute 1 (*AGO1*)	-1.36^a^	2.87	9.82E-02	9.10E-07
SRY-box transcription factor 9 (*Sox-9*)	NA	2.31	NA	1.36E-11
Neuroparsin (*NP*)	-2.21^a^	1.87	1.10E-03	7.55E-05
Profilin	-2.72	1.71	8.39E-19	0.00
Prostaglandin E synthase 2 (*Pges2*)	0.51^b^	1.41	4.96E-09	1.20E-88
Protein fem-1 homolog A (*FEM-1a*)	-0.84^b^	1.37	8.97E-06	3.20E-06

NA indicates that no transcriptome data were found.

^a^ gene expression is not different between the testis and ovary because FDR > 0.001.

^b^ gene expression is not different between the testis and ovary because the absolute value of log_2_fold-change is less than 1.

### Validation of transcriptome analysis using qPCR

We used qPCR to validate the transcriptome data. The expressions of *dsx* and *FoxL2* were selected as representative DEGs for testis development. Additionally, *SOP1* and *Vtg* were used to validate the transcriptome data in relation to ovarian development. The genes selected for qPCR were based on early and late gonad development requirements.

**Fig 4:**
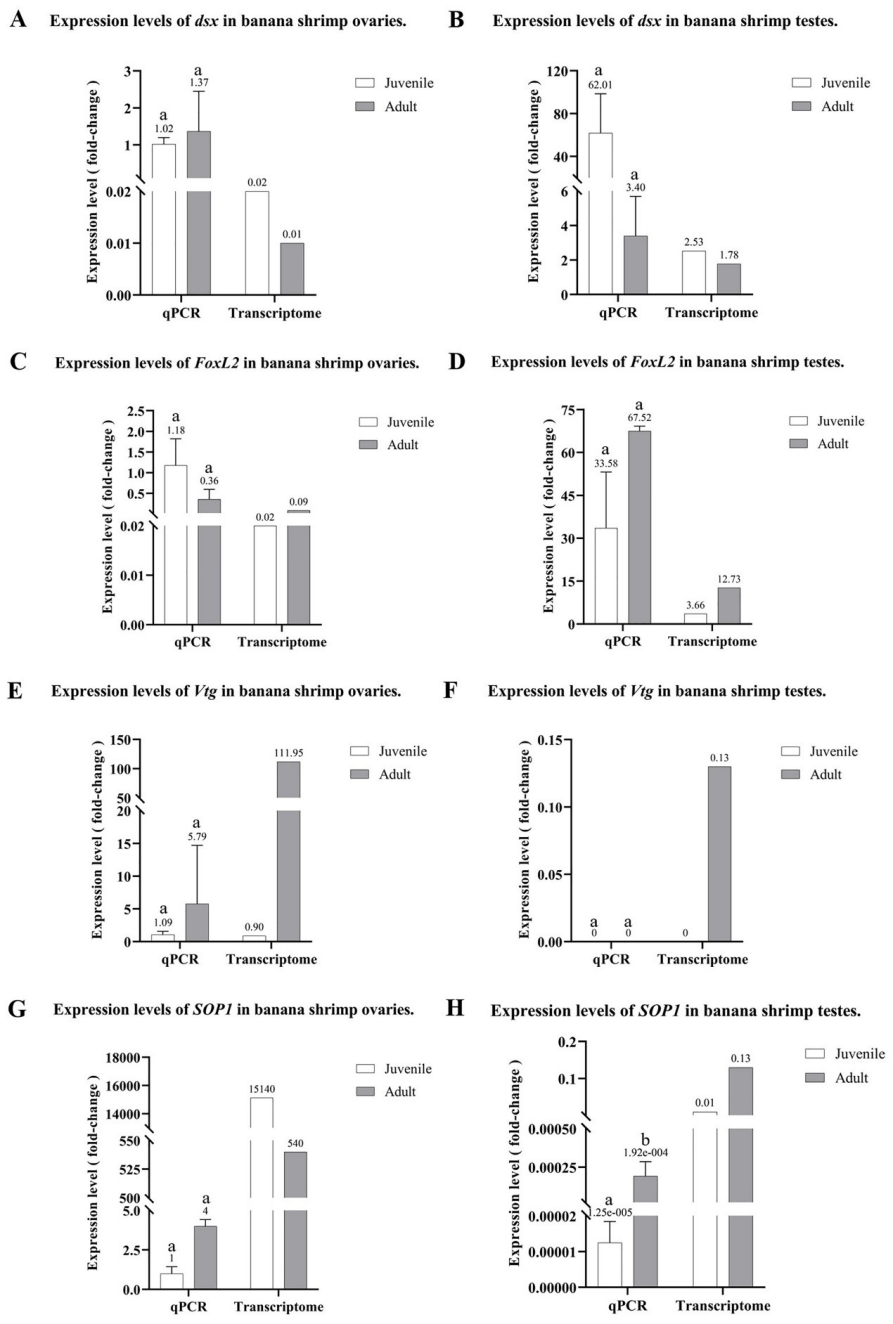
Validation of transcriptome data using qPCR. Fold-change in gene expression using transcriptome data and qPCR. Fold changes represent gene expression in the ovary and testis of shrimp in the juvenile and adult stages (n = 3). (A) and (B) show *dsx* expressions. (C) and (D) indicate *FoxL2* expression. (E) and (F) show *Vtg* expression levels. (G) and (H) show *SOP1* expression. Different letters (a, b) indicate a significant expression between sample groups at a *p-value* < 0.05.

The expression of *dsx* in the testis and ovary of banana shrimp was evaluated in Fig 4A and 4B. The *dsx* expression level was higher in the banana shrimp testis than that of the ovary and showed lower expression levels in adult testis compared to that of juveniles. These findings suggest that *dsx* plays a more important role in male sexual development. qPCR analysis revealed that *FoxL2* gene expression was relatively elevated in the testis when compared to that of the ovary and exhibited higher expression levels in adult testis when compared to that of juveniles (Fig 4C and 4D).

*Vtg* plays a role in ovary development in female banana shrimp. *Vtg* was not expressed in the testis samples (both juvenile and adult) but at high levels in adult ovary samples (Fig 4E and 4F). The gene *SOP1* was also found to be more important in adult ovary development, as higher expression levels were observed in adult ovary samples when compared to those of testis in both qPCR and transcriptome data (Fig 4G and 4H). The expression levels of *dsx*, *FoxL2*, *Vtg*, and *SOP1* obtained from qPCR showed the same pattern as the transcriptome data.

### Sequence and phylogenetic analysis of *FoxL2*

**Fig 5:**
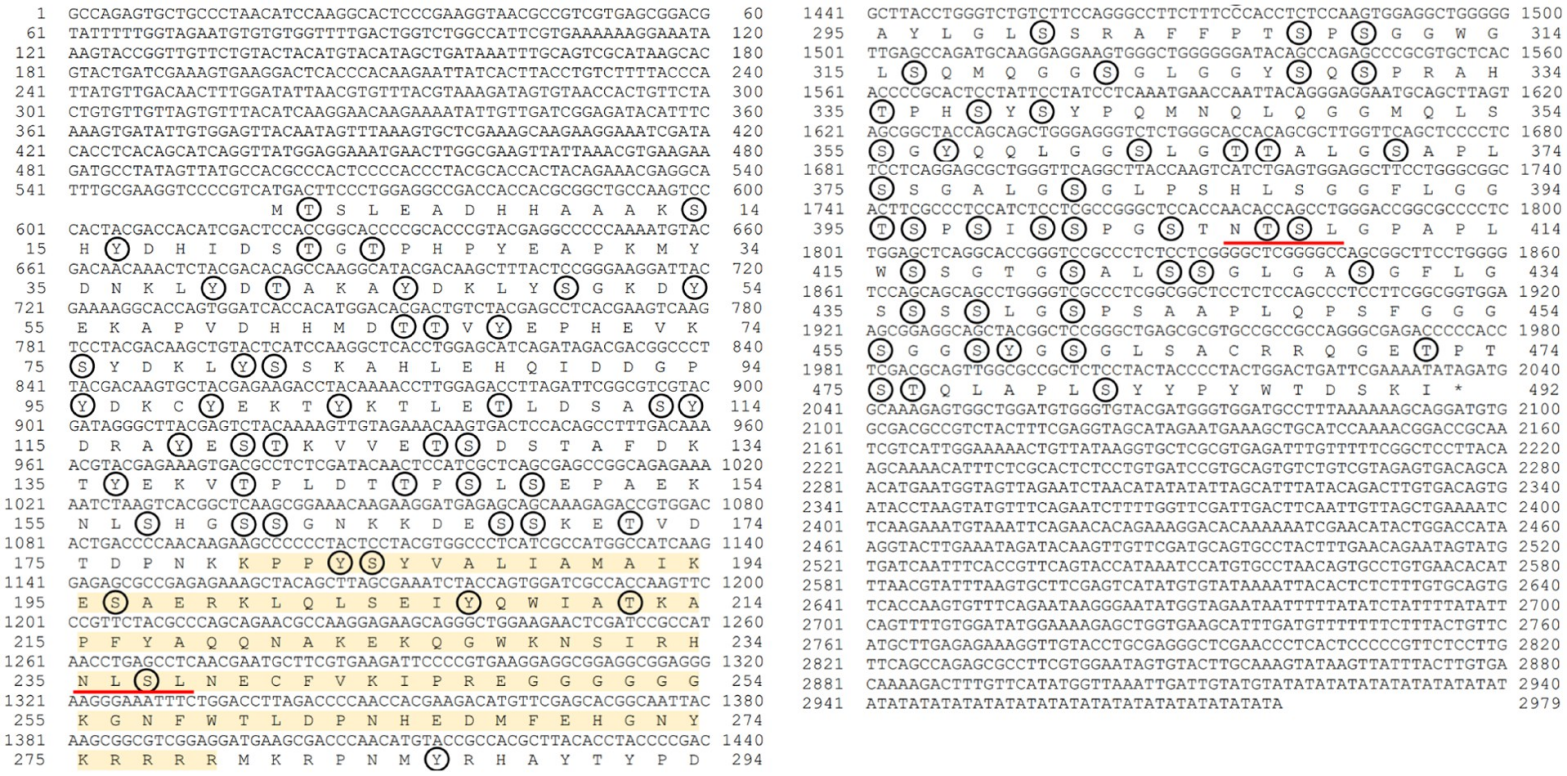
Nucleotide and amino acid sequences of *F*. *merguiensis FoxL2*. The *FoxL2* domain is depicted in yellow. The red line underlines two glycosylation sites, and phosphorylation sites are circled in black.

**Fig 6:**
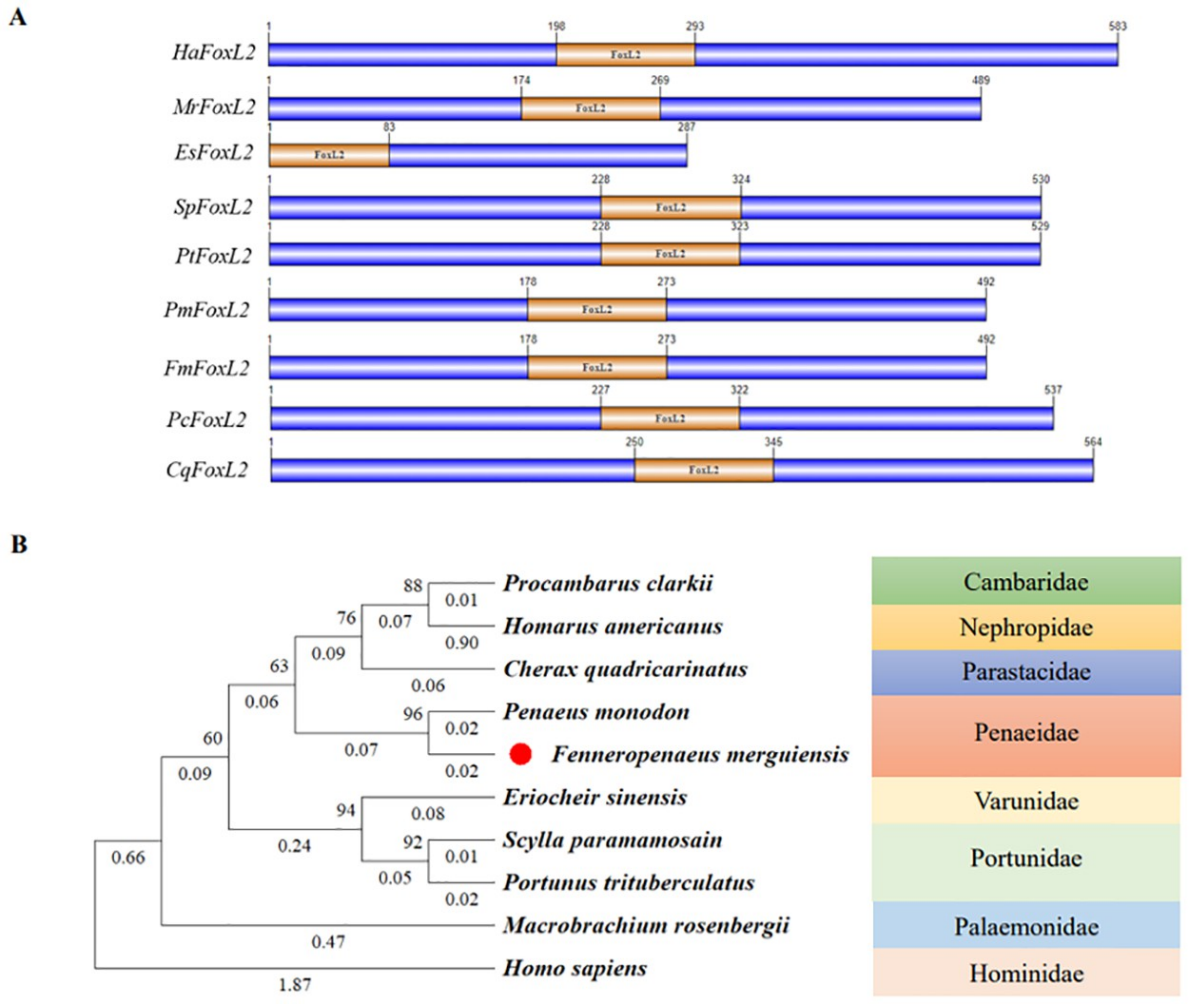
Diagram of the *FoxL2* domain and phylogenetic analysis of FoxL2 proteins in banana shrimp and other species. (A) The chart of the *FoxL2* domain according to species. Ha: *H*. *americanus*; Mr: *M*. *rosenbergii*; Es: *E*. *sinensis*; Sp: *S*. *paramamosain*; Pt: *P*. *trituberculatus*; Pm: *P*. *monodon*; Fm: *F*. *merguiensis*; Pc: *P*. *clarkii*; Cq: *C*. *quadricarinatus*. (B) The neighbor-joining phylogenetic tree of *FoxL2* amino acid sequences from different organisms was constructed by MEGA 11.0, and 1,000 bootstrap replicates were used to assess the confidence in each node.

Since *FoxL2* was more highly expressed in the testis than the ovary in shrimp, and *FoxL2* is involved in cell proliferation and differentiation during human ovarian development [12, 13]. Therefore, the full-length cDNA of the banana shrimp *FoxL2* was analyzed from transcriptome data. It has 1,497 base pairs, encodes 492 amino acids with 53.2 kDa, and has an isoelectric point (pI) of 6.81. The *FoxL2* sequence contains two glycosylation sites and 87 phosphorylation sites (Fig 5). The forkhead domain is spaned from 180 to 279 (Fig 5). *FoxL2* belongs to the winged helix/forkhead transcription factor family, which consists of a conserved DNA-binding domain known as the forkhead box [57]. The *FoxL2* identities of eight different decapod species (*Homarus americanus*, *M*. *rosenbergii*, *E*. *sinensis*, *S*. *paramamosain*, *Portunus trituberculatus*, *P*. *monodon*, *Procambarus clarkii*, *Cherax quadricarinatus*, *and F*. *merguiensis)* were compared. The *FoxL2* domain of *F*. *merguiensis* is the same size (96 amino acids) as *P*.*monodon*, and the *FoxL2* domain area is in the same position (Fig 6A). *S*. *paramamosain* has the longest *FoxL2* domain size (97 amino acids). In addition, *E*. *sinensis* has the smallest domain size (~84 amino acids), while other species share a common domain size, including *F*. *merguiensis* (~96 amino acids).

The highest nucleic acid sequence identity of *FoxL2* was 95.93%, which was shared with *P*. *monodon*, and the lowest was 62.73% with *M*. *rosenbergii* (S1 Fig). Phylogenetic analysis revealed that the *FoxL2* proteins of *F*. *merguiensis* were most closely related to those of *P*. *monodon* (Fig 6B). The amino acid sequence of the forkhead domain between *F*. *merguiensis* and *P*. *monodon* is 100% identical. Comparisons between *S*. *paramamosain* and *F*. *merguiensis* showed 89.58% similarity in the forkhead domain, where the conserved domain was identical.

### Prediction of the FoxL2 protein *Vtg* promoter binding site

**Fig 7:**
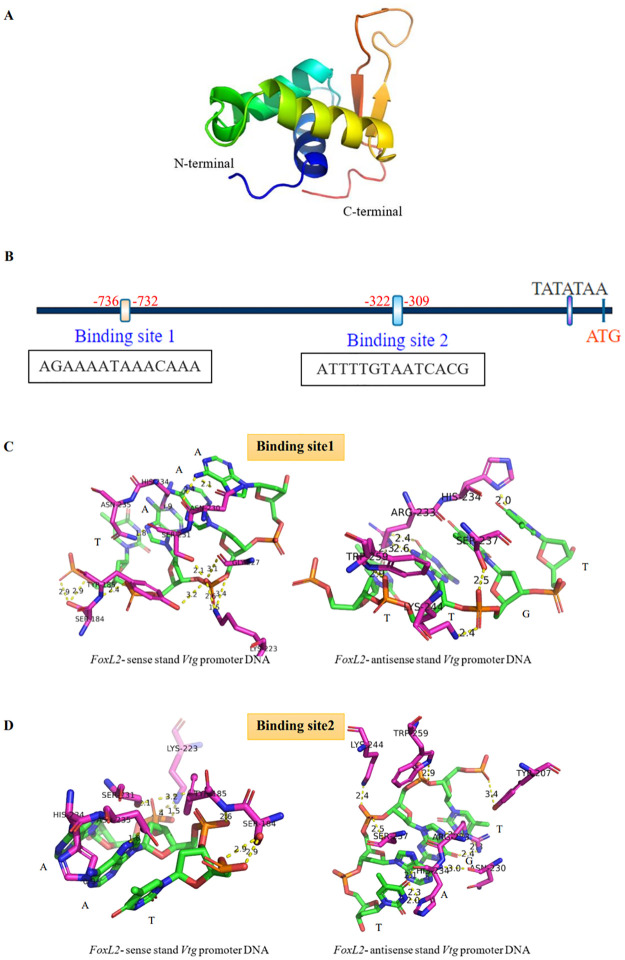
A model of the three-dimensional structure of *F*. *merguiensis* FoxL2 binding to the *Vtg* promoter. (A) The three-dimensional structure of the FoxL2 domain of *F*. *merguiensis*. (B) Two nucleotide-binding regions bind to the FoxL2 proteins, predicted using the JASPAR database. (C) The first binding site and (D) the second binding site in the FoxL2 domain (pink) connect with the promoter segment of the *Vtg* gene in *S*. *paramamosain* (green).

Before experimenting with an animal model, the FoxL2 protein was screened for binding to the *Vtg* promoter using *in silico* binding. A three-dimensional protein structure model of the FoxL2 forkhead domain was constructed and contained three alpha helices and two beta sheets (Fig 7A). The appearance of such a three-dimensional structure follows the winged helix, which is characteristic of the forkhead box domain. The model had a QMEANDisCo global score of 0.63 ± 0.09 and a sequence identity of 76.09% to human FoxL2 (PDB ID: 7vou.1.C). The JASPAR program was used to predict binding sites between the *FoxL2* domain of *F*. *merguiensis* and the *Vtg* promotor of *S*. *paramamosain*. A total of five positions were predicted. The top two binding positions with the highest relative scores (rs) were 5′-AGAAAATAAACAAA-3′ (rs: 0.89) and 5′-ATTTTGTAATCACG-3′ (rs: 0.84) (Fig 7B). The second binding position is consistent with the prediction of FoxL2 binding to the *Vtg* promoter of *S*. *paramamosain* [33].

The model illustrates the interaction site between FoxL2 and the *Vtg* promoter, with the two positions depicted in Fig 7C and 7D, respectively. Predictions show that FoxL2 at SER184, TYR185, LYS223, SER231, HIS234, and ASN235 binds to two DNA binding sites of the *Vtg* promoter through hydrogen bonds or other types of electrostatic interactions. The first binding site has the shortest distance between the nitrogen atom of HIS234 and the nitrogen atom at the second position of the phosphate group of adenine-729 of the *Vtg* promotor, which is 0.9 angstroms. Meanwhile, FoxL2 at ARG233, HIS234, SER237, LYS244, and TRP259 binds to the first antisense DNA binding site with the shortest distance is 2.0 angstroms. The first antisense site is between the nitrogen atom of HIS234 and the nitrogen atom located at the third position in the nucleotide sugar ring of thymine, which is complementary to adenine-731 of the *Vtg* promotor. In addition, the identical amino acid residues of FoxL2 bound with the second *Vtg* promoter binding site. The minimum distance recorded at this binding site is 0.9 angstroms, particularly between HIS234’s nitrogen atom and the primary position nitrogen atom of adenine-315 within the *Vtg* promoter.

Furthermore, FoxL2 at TYR207, ASN230, ARG233, HIS234, SER237, LYS244, and TRP259 are associated with the antisense DNA strand using hydrogen bonds or other electrostatic interactions. This specific location is the shortest at 2.0 angstroms, directly linking the nitrogen atom of HIS234 with the nitrogen atom at three specific positions within the nucleotide sugar ring of thymine. This particular interaction corresponds with adenine-314 of the *Vtg* promoter.

### Effects of *FoxL2* knock-down in female banana shrimp

**Fig 8:**
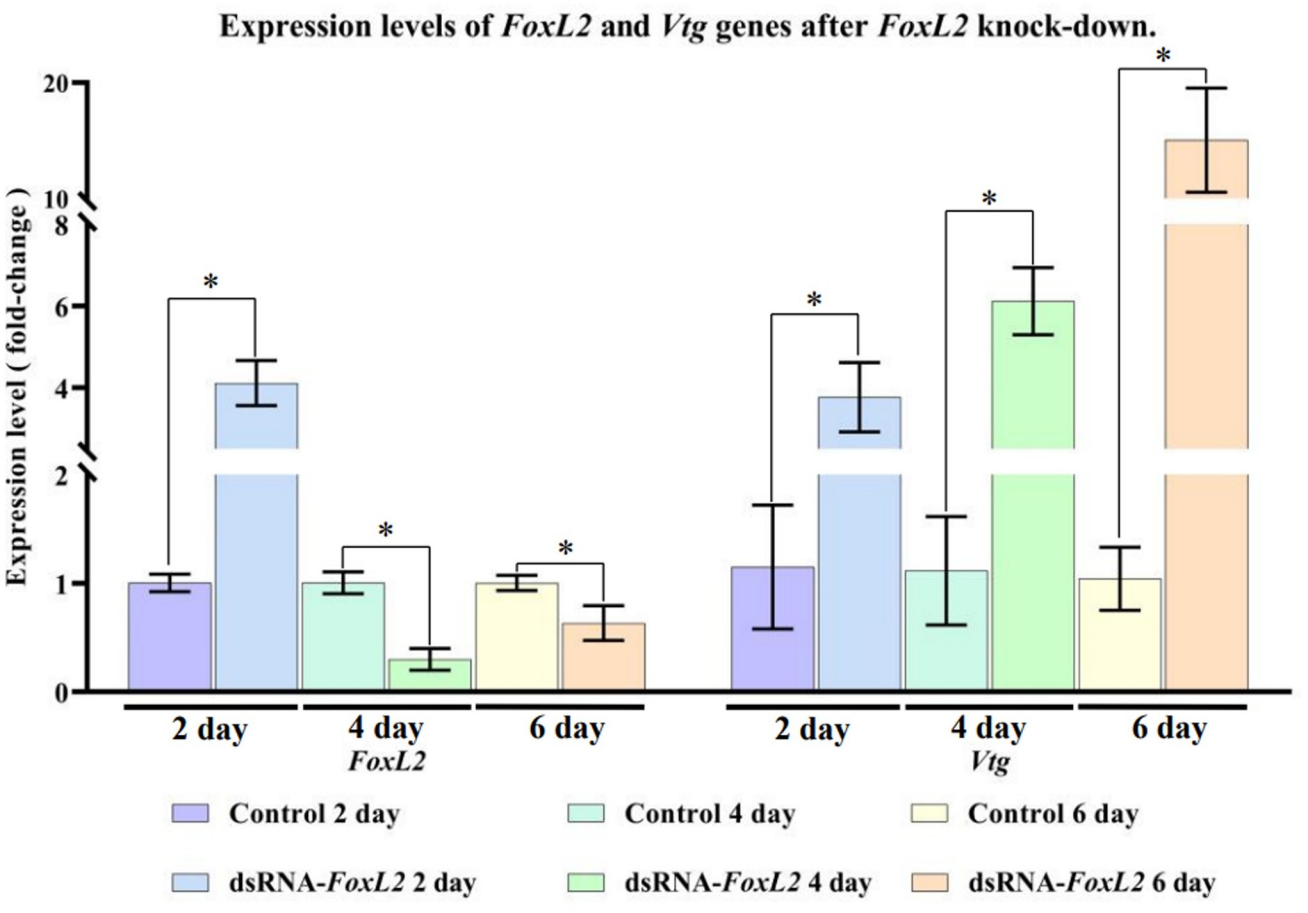
Expression levels of *FoxL2* and other ovarian genes after *FoxL2* knock-down. Expression levels of *FoxL2* and *Vtg* genes at 2, 4, and 6 d post-injection (dsRNA-*FoxL2*) were analyzed using three replicates. The symbol * indicates a significant difference between sample groups, *p-value* < 0.05.

**Fig 9:**
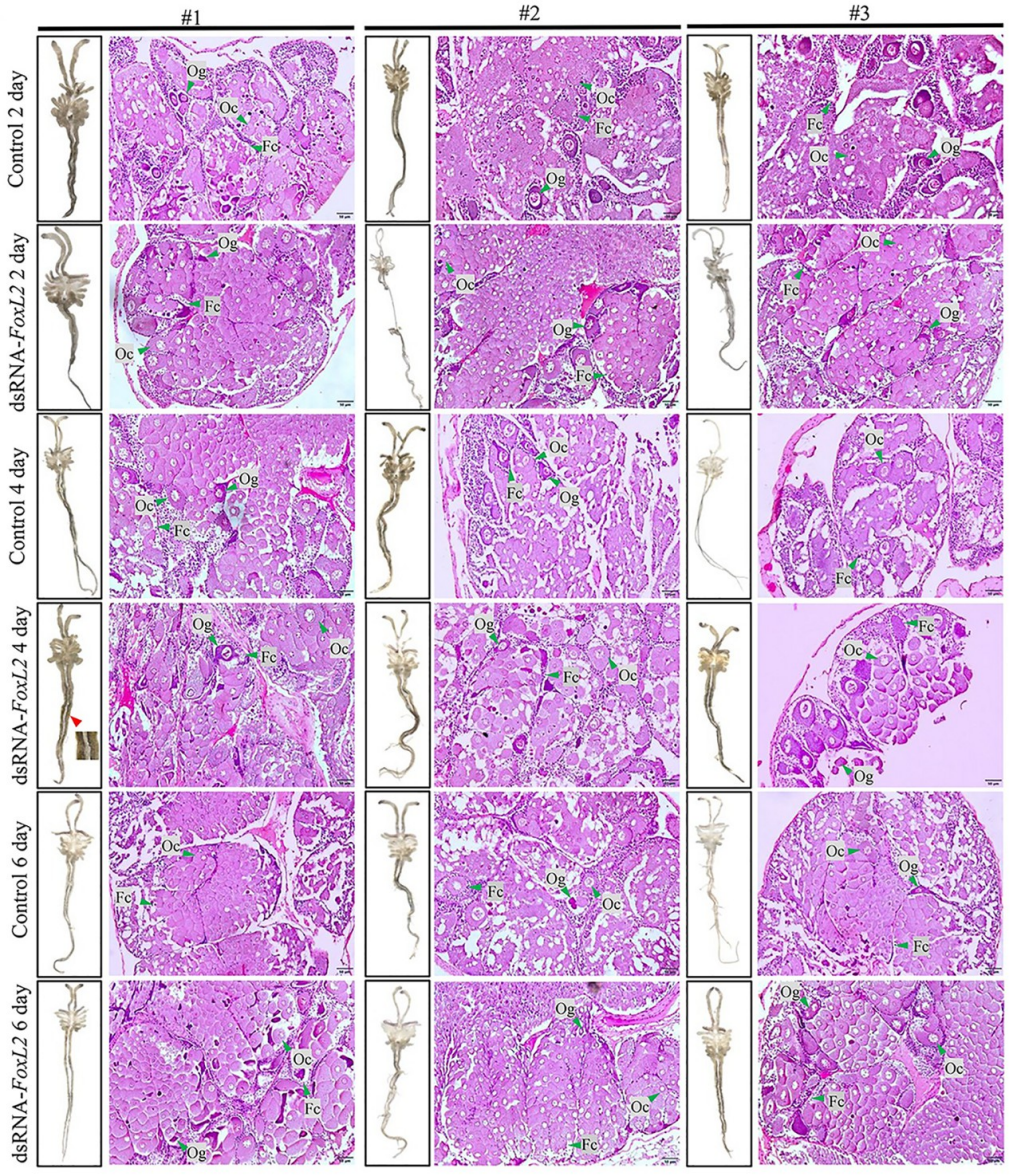
Morphology and Histological investigation of ovarian samples after *FoxL2* knock-down. The ovarian samples after *FoxL2* knock-down experiment were sectioned (5μmthick) to determine ovarian cell development after downregulation of *FoxL2* expression. Red arrows indicate areas where ovary discoloration to yellow was observed. Og: Oogonia; Oc: oocytes; Fc: follicular cells. The tissue image was taken at 20X magnification under a BX-53 light microscope equipped with a DP-72 digital camera (Olympus, Tokyo, Japan).

As a positive result of in silico *Vtg* promoter binding, *FoxL2* knocked-down in female banana shrimp was performed. After dsRNA-*FoxL2* was injected at 2, 4, and 6 d, the expression level of *FoxL2* genes was significantly down-regulated than those of the control group. While the expression levels of *Vtg* were found to be significantly higher (*p-value* < 0.05) than those of the control group at 2, 4, and 6 d post-injection with dsRNA-*FoxL2* (Fig 8). In addition, the morphology and histology of the ovaries after down-regulated *FoxL*2 expression were overall developed, corresponding to the increase of the *Vtg* expression, shown in Fig 9. These results indicated that *FoxL2* expression was successfully knocked down by dsRNA-*FoxL2*. Meanwhile, *Vtg* expression was activated when *FoxL2* was reduced.

## Discussion

*F*. *merguiensis* is popular for consumer and commercial species in several countries, including Southeast Asian countries, India, and Australia. Aquaculture of this species may economically benefit these countries in terms of food security and income. Understanding the reproductive system of this shrimp is essential for aquaculture applications. Although the RNA-seq approach has been reported for *F*. *merguiensis* [4, 6, 10, 11] and other shrimps, more information focusing on sexual and reproductive development is required to increase seed quality and obtain sustainable cultures. This study used transcriptome analysis to investigate the dynamic change in gene expression between juvenile and adult ovaries and testes. This study revealed that the detected unigenes in the juvenile testes and ovaries were about 50% of those in the adult stage. Interestingly, the total number of DEGs (unigenes) in the testis was 36.2% and 5.2% in juvenile and adult testis, respectively. In contrast, the total number of DEGs in the ovary was 9.5% and 31.4% in the juvenile and adult stages, respectively (Table 2). This indicates that the development in the adult ovary required more upregulated genes than in the adult testis. A comparison between the ovary and testis transcriptome data was also reported in *P*. *serratus* and demonstrated that genes were more upregulated in the adult ovary when compared to the adult testis [4], which is similar to the data shown in this study.

In this study, histological techniques after H&E staining of ovarian tissue were classified into 4 stages as previous reports: undeveloped stage (oocyte<65 in diameter), early development (stage I, oocyte 75–125 in diameter)), developing (stage II, oocyte 100–200 μm in diameter, fat droplet, cortical rods) and mature (stage III, oocyte 125–250 μm in diameter, fat droplet, cortical rods) [58–61]. In this study, the H&E staining juvenile’s ovarian sample contained oogonia, primary oocyte (<65 μm in diameter), and primary follicle cell was classified as undeveloped ovary stage. The adult’s ovarian sample comprised of oogonia, oocyte (75–125 μm in diameter), and primary follicle cell, defined as early development stage I (Fig 1). Therefore, the expressed genes in the adult’s ovarian sample were more than in the juvenile’s ovarian sample. In the juvenile ovary, meiosis occurs to accumulate arrested oocytes in previtellogenic growth. A secondary oocyte maturation (vitellogenin) takes place at the adult stage [62]. Then the secondary oocyte maturation (vitellogenin) resumes meiosis by the time spawning. It corresponds with the DEGs that were shared both in the juvenile and adult stages. The *SOP1*, *SOP2*, *Ptgr1*, *SOP*, *Vmo1*, *Tra-2c*, *EST*, *Vgr*, *TR-beta A* genes. Meanwhile, the DEGs of *Vtg*, *JHE1*, and *Pxt* genes were significantly found in the adult ovary.

In this study, the *SOP* of *F*. *merguiensis* was the high expressed during the juvenile (early previtellogenic stage, which corresponds with the expression of the *SOP* of *Masupenaeus japonicus*. The *SOP* of *M*. *japonicus* in the ovary was higher in the previtellogenic stage than the endogenic stage, early exo-vitellogenic stage, late exo-vitellogenic stage and mature stage [63]. The same pattern of *Vtg* mRNA in the ovary was reported to slightly increase in the previtellogenic stage and higher expression in the endogenic stage than in the early, late, and mature stages. In contrast, the *Vtg* expressed in the hepatopancreas at a low level during the previtellogenic to the endogenic stage, increased the level in the exo-vitellogenic stage and the highest level in the mature stage. *SOP* and *Vtg* were also produced from extra-ovary (hepatopancreas) in *M*. *japonicus*, *P*. *vannamei*, *P*. *semisulcatus*, *P*. *monodon* [63–66]. *Vtg* of *F*. *merquiensis* is initially upregulated expression in the previtellogenic in this study and in the previous study [67] and is the highest expression in the vitellogenin stage [60].

Vmo*1* is one of the essential proteins in the ovary and mRNA was detected in the previtellogenic ovary in this study. In comparison, *Vmo1* was not found in the ovary of *L*. *vannamei* and was reported only produced from the hepatopancreas during vitellogenesis and imported into the ovary [55]. The different stages and species of shrimp provided different sources of protein in the ovary. For example, *Vtg* of *M*. *rosenbergii* was produced only from hepatopancreas [68], while *Vtg of M*. *japonicus*, *P*. *vannamei*, *P*. *semisulcatus*, *P*. *monodon* was from ovary and hepatopancreas [63–66].

Testis, where spermatogenesis occurs and comprises spermatocytogenesis and spermiogenesis stages. The spermatocytogenesis produces haploid spermatids from diploid spermatogonia. Spermiogenesis is the process that transforms the mature spermatids into spermatozoa [69]. Due to the juvenile testis sample containing most spermatogonia and primary spermatocyte to transform into spermatid, many expressed genes were found. While the adult testis sample consisted of many spermatids, the primary process is spermiogenesis, and fewer genes were expressed in the adult testis than in the juvenile testis (Figs 1 and 2). Additionally, this study was supported by the report that DNA synthesis in testes at the pre-molt stage is higher than at the post-molt and inter-molt stages in *M*. *rosenbergii* [70, 71]. However, the spermatogenesis in *L*. *vannamei* is continuous without related to the molt cycle [72].

Since *FoxL2* is known to be involved in female development in humans and mice, the suppression of *Sox-*9 expression in humans and mice suggests that the *FoxL2* gene plays a role in regulating cell differentiation and proliferation [20]. *FoxL2* is involved in the synthesis of estrogen and aromatase in mammals and fish [22–26]. In *F*. *merguiensis*, transcriptome analysis and qPCR showed that the *FoxL2* gene was expressed in the juvenile testis and was upregulated as it progressed into the adult stage (Fig 2C and 2D). The reason for the higher expression of *FoxL2* in the testis than in the ovary of the shrimp remains unclear. *FoxL2* gene expression in *M*. *rosenbergii* was reported to be highest in males (zz), followed by females (zw) and super females (ww) [73]. Moreover, the expression pattern of *FoxL2* was analyzed using fluorescence *in situ* hybridization in *S*. *paramamosain*, which revealed signals in both the ovary and testis. In the ovary, signals were identified in follicle cells. In contrast, in the testis, signals were detected in the epithelium of the seminiferous tubules, spermatogonia, spermatocytes, and spermatids. This suggests that *FoxL2* may be crucial in testis differentiation and development [74].

In crustacean species, *E*. *sinensis* [30] and *S*. *paramamosain* [33] showed high expression levels of *Vtg* in the ovary, while low expression of *FoxL2* was found. In addition, a *FoxL2* mechanism was proposed to bind to *Ftz-F1*, inhibiting *Ftz-F1* from binding to the cytochrome P450 promoter and suppressing *Vtg* expression [30]. Moreover, it has been reported that *FoxL2* inhibits *Vtg* expression by binding the *Vtg* promoter in *S*. *paramamosain* [33]. In the transcriptome analysis of this study, the *Ftz-F1* and *FoxL2* transcripts were higher in both the juvenile and adult testes when compared to those of the ovary (Table 3), which may support the *FoxL2* mechanism affects *Vtg* expression in *F*. *merguiensis*. In addition, knock-down by the dsRNA injection significantly suppressed *FoxL2* when compared to the control 2, 4, and 6 d post-injection (Fig 8). Consequently, *Vtg* expression was activated when compared between 2, 4, and 6 d post-injection. These phenomena indicate that *FoxL2* reduction is essential for *Vtg* expression. Further investigation is required to confirm mechanism of these findings.

## Supporting information

S1 Fig Multiple alignments of the deduced amino acid sequence of the *FoxL2* gene. Multiple alignments of the deduced amino acid sequence of the *FoxL2* gene, GenBank accession no. OQ870552, with *H*. *americanus*: accession no. KAG7154875.1, *M*. *rosenbergii*: accession no. USJ75257.1, *E*. *sinensis*: accession no. AIS92518.1, *S*. *paramamosain*: accession no. QQY98966.1, *P*. *trituberculatus*: accession no. WAA68168.1, *P*. *monodon*: accession no. XP_037795163.1, *P*. *clarkii*: accession no. ALD48735.1, and *C*. *quadricarinatus*: accession no. UWX37250.1. The red boxes represent the *FoxL2* conserved domain.
(TIF)

## References

[1] Franco AR, Ferreira JG, Nobre AM. Development of a growth model for penaeid shrimp. Aquaculture. 2006;259: 268–277. doi: 10.1016/J.AQUACULTURE.2006.05.051

[2] Gomelsky B. Chromosome set manipulation and sex control in common carp: a review. Aquat Living Resour. 2003;16: 408–415. doi: 10.1016/S0990-7440(03)00085-8

[3] Li S, Li F, Wen R, Xiang J. Identification and characterization of the sex-determiner Transformer-2 homologue in Chinese shrimp, *Fenneropenaeus chinensis*. Sex Dev. 2012;6: 267–278. doi: 10.1159/000341377 22846336

[4] González-castellano I, Manfrin C, Pallavicini A, Martínez-lage A. De novo gonad transcriptome analysis of the common littoral shrimp Palaemon serratus: novel insights into sex-related genes. BMC Genomics. 2019;4: 1–15. doi: 10.1186/s12864-019-6157-4 31640556 PMC6805652

[5] Velázquez-Lizárraga AE, Juárez-Morales JL, Racotta IS, Villarreal-Colmenares H, Valdes-Lopez O, Luna-González A, et al. Transcriptomic analysis of Pacific white shrimp (*Litopenaeus vannamei*, Boone 1931) in response to acute hepatopancreatic necrosis disease caused by *Vibrio parahaemolyticus*. PLoS One. 2019;14: 1–28. doi: 10.1371/journal.pone.0220993 31408485 PMC6692014

[6] Peng J, Wei P, Zhang B, Zhao Y, Zeng D, Chen X, et al. Gonadal transcriptomic analysis and differentially expressed genes in the testis and ovary of the Pacific white shrimp (*Litopenaeus vannamei*). BMC Genomics. 2015; 1–18. doi: 10.1186/s12864-015-2219-4 26607692 PMC4659196

[7] Vlasova-St. Louis I, Gorzalski A, Pandori M. Diagnostic Applications for RNA-Seq Technology and Transcriptome Analyses in Human Diseases Caused by RNA Viruses. Applications of RNA-Seq in Biology and Medicine. 2016. pp. 225–240. doi: 10.5772/intechopen.99156

[8] Iñiguez LP, Ramírez M, Barbazuk WB, Hernández G. Identification and analysis of alternative splicing events in Phaseolus vulgaris and Glycine max. BMC Genomics. 2017;18: 1–17. doi: 10.1186/s12864-017-4054-2 28830361 PMC5568362

[9] Valenzuela-miranda D, Gallardo-escárate C, Valenzuela-muñoz V, Farlora R, Gajardo G. Sex-dependent transcriptome analysis and single nucleotide polymorphism (SNP) discovery in the brine shrimp *Artemia franciscana*. Mar Genomics. 2014;18: 151–154. doi: 10.1016/j.margen.2014.10.007 25450167

[10] Powell D, Knibb W, Remilton C, Elizur A. De-novo transcriptome analysis of the banana shrimp (*Fenneropenaeus merguiensis*) and identification of genes associated with reproduction and development. Mar Genomics. 2015;22: 71–78. doi: 10.1016/j.margen.2015.04.006 25936497

[11] Yan H, Cui X, Shen X, Wang L, Jiang L, Liu H, et al. De novo transcriptome analysis and differentially expressed genes in the ovary and testis of the Japanese mantis shrimp *Oratosquilla oratoria* by RNA-Seq. Comp Biochem Physiol Part D Genomics Proteomics. 2018;26: 69–78. doi: 10.1016/j.cbd.2018.04.001 29702368

[12] Uhlenhaut NH, Treier M. Foxl2 function in ovarian development. Mol Genet Metab. 2006;88: 225–234. doi: 10.1016/j.ymgme.2006.03.005 16647286

[13] Uhlenhaut NH, Treier M. Forkhead transcription factors in ovarian function. Reproduction. 2011;142: 489–495. doi: 10.1530/REP-11-0092 21810859

[14] Georges A, Auguste A, Bessière L, Vanet A, Todeschini AL, Veitia RA. FOXL2: a central transcription factor of the ovary. J Mol Endocrinol. 2014;52: R17–R33. doi: 10.1530/JME-13-0159 24049064

[15] Ellsworth BS, Egashira N, Haller JL, Butts DL, Cocquet J, Clay CM, et al. FOXL2 in the pituitary: Molecular, genetic, and developmental analysis. Mol Endocrinol. 2006;20: 2796–2805. doi: 10.1210/me.2005-0303 16840539

[16] Corpuz PS, Lindaman LL, Mellon PL, Coss D. FoxL2 is required for activin induction of the mouse and human follicle-stimulating hormone β-subunit genes. Mol Endocrinol. 2010;24: 1037–1051. doi: 10.1210/me.2009-0425 20233786 PMC2870942

[17] Tran S, Zhou X, Lafleur C, Calderon MJ, Ellsworth BS, Kimmins S, et al. Impaired fertility and FSH synthesis in gonadotrope-specific Foxl2 knockout mice. Mol Endocrinol. 2013;27: 407–421. doi: 10.1210/me.2012-1286 23340250 PMC3589670

[18] Cocquet J, Pailhoux E, Jaubert F, Servel N, Xia X, Pannetier M, et al. Evolution and expression of FOXL2. J Med Genet. 2002;39: 916–921. doi: 10.1136/jmg.39.12.916 12471206 PMC1757225

[19] Auguste A, Chassot AA, Grégoire EP, Renault L, Pannetier M, Treier M, et al. Loss of R-spondin1 and Foxl2 amplifies female-to-male sex reversal in XX mice. Sex Dev. 2012;5: 304–317. doi: 10.1159/000334517 22116255

[20] Ottolenghi C, Omari S, Garcia-Ortiz JE, Uda M, Crisponi L, Forabosco A, et al. Foxl2 is required for commitment to ovary differentiation. Hum Mol Genet. 2005;14: 2053–2062. doi: 10.1093/hmg/ddi210 15944199

[21] Ottolenghi C, Pelosi E, Tran J, Colombino M, Douglass E, Nedorezov T, et al. Loss of Wnt4 and Foxl2 leads to female-to-male sex reversal extending to germ cells. Hum Mol Genet. 2007;16: 2795–2804. doi: 10.1093/hmg/ddm235 17728319

[22] Pannetier M, Fabre S, Batista F, Kocer A, Renault L, Jolivet G, et al. FOXL2 activates P450 aromatase gene transcription: Towards a better characterization of the early steps of mammalian ovarian development. J Mol Endocrinol. 2006;36: 399–413. doi: 10.1677/jme.1.01947 16720712

[23] Fleming NI, Knower KC, Lazarus KA, Fuller PJ, Simpson ER, Clyne CD. Aromatase is a direct target of FOXl2: C134W in granulosa cell tumors via a single highly conserved binding site in the ovarian specific promoter. PLoS One. 2010;5. doi: 10.1371/journal.pone.0014389 21188138 PMC3004790

[24] Batista F, Vaiman D, Dausset J, Fellous M, Veitia RA. Potential targets of FOXL2, a transcription factor involved in craniofacial and follicular development, identified by transcriptomics. Proc Natl Acad Sci U S A. 2007;104: 3330–3335. doi: 10.1073/pnas.0611326104 17360647 PMC1805535

[25] Wang DS, Kobayashi T, Zhou LY, Paul-Prasanth B, Ijiri S, Sakai F, et al. Foxl2 up-regulates aromatase gene transcription in a female-specific manner by binding to the promoter as well as interacting with Ad4 binding protein/steroidogenic factor. Mol Endocrinol. 2007;21: 712–725. doi: 10.1210/me.2006-0248 17192407

[26] Rosario R, Araki H, Print CG, Shelling AN. The transcriptional targets of mutant FOXL2 in granulosa cell tumours. PLoS One. 2012;7: 1–11. doi: 10.1371/journal.pone.0046270 23029457 PMC3460904

[27] Zhang X, Li M, Ma H, Liu X, Shi H, Li M, et al. Mutation of foxl2 or cyp19a1a results in female to male sex reversal in XX nile tilapia. Endocrinology. 2017;158: 2634–2647. doi: 10.1210/en.2017-00127 28838139

[28] Fan Z, Zou Y, Liang D, Tan X, Jiao S, Wu Z, et al. Roles of forkhead box protein L2 (foxl2) during gonad differentiation and maintenance in a fish, the olive flounder (*Paralichthys olivaceus*). Reprod Fertil Dev. 2019;31: 1742–1752. doi: 10.1071/RD18233 31537253

[29] Gan RH, Wang Y, Li Z, Yu ZX, Li XY, Tong JF, et al. Functional Divergence of Multiple Duplicated Foxl2 Homeologs and Alleles in a Recurrent Polyploid Fish. Mol Biol Evol. 2021;38: 1995–2013. doi: 10.1093/molbev/msab002 33432361 PMC8097289

[30] Li Q, Xie J, He L, Wang Y, Yang H, Duan Z, et al. FOXL2 down-regulates vitellogenin expression at mature stage in *Eriocheir sinensis*. Biosci Rep. 2015;35(6): 1–17. doi: 10.1042/BSR20150151 26430246 PMC4708011

[31] Chimnual J, Saetan J, Chotigeat W. Histological demonstration of gonad development in the banana shrimp, *Fenneropenaeus merguiensis*. Aquac Fish. 2023. doi: 10.1016/j.aaf.2023.06.003

[32] Livak KJ, Schmittgen TD. Analysis of relative gene expression data using real-time quantitative PCR and the 2^-ΔΔCT^ method. Methods. 2001;25: 402–408. doi: 10.1006/meth.2001.1262 11846609

[33] Wan H, Zhong J, Zhang Z, Xie Y, Wang Y. Characterization of the foxl2 gene involved in the vtg expression in mud crab (*Scylla paramamosain*). Gene. 2021;798. doi: 10.1016/j.gene.2021.145807 34224832

[34] Wei WY, Huang JH, Zhou FL, Yang Q Bin, Li YD, Jiang S, et al. Identification and expression analysis of Dsx and its positive transcriptional regulation of IAG in black tiger shrimp (Penaeus monodon). Int J Mol Sci. 2022;23. doi: 10.3390/ijms232012701 36293554 PMC9604489

[35] Wei WY, Huang JH, Yang Q Bin, Zhou FL, Jiang S, Li YD, et al. Molecular characterization and functional analysis of DMRT11E in black tiger shrimp (*Penaeus monodon*). Aquac Reports. 2022;22: 100982. doi: 10.1016/J.AQREP.2021.100982

[36] Li S, Li F, Yu K, Xiang J. Identification and characterization of a doublesex gene which regulates the expression of insulin-like androgenic gland hormone in *Fenneropenaeus chinensis*. Gene. 2018;649: 1–7. doi: 10.1016/J.GENE.2018.01.043 29339074

[37] Moschall R, Rass M, Rossbach O, Lehmann G, Kullmann L, Eichner N, et al. Drosophila Sister-of-Sex-lethal reinforces a male-specific gene expression pattern by controlling Sex-lethal alternative splicing. Nucleic Acids Res. 2019;47: 2276–2288. doi: 10.1093/nar/gky1284 30590805 PMC6411925

[38] Guo Q, Li S, Lv X, Xiang J, Sagi A, Manor R, et al. A putative insulin-like androgenic gland hormone receptor gene specifically expressed in male Chinese shrimp. Endocrinology. 2018;159: 2173–2185. doi: 10.1210/en.2017-03253 29596627

[39] Tan K, Jiang H, Jiang D, Wang W. Sex reversal and the androgenic gland (AG) in *Macrobrachium rosenbergii*: A review. Aquac Fish. 2020;5: 283–288. doi: 10.1016/j.aaf.2019.11.004

[40] Galindo-Torres P, Ventura-López C, Llera-Herrera R, Ibarra AM. A natural antisense transcript of the fem-1 gene was found expressed in female gonads during the characterization, expression profile, and cellular localization of the fem-1 gene in Pacific white shrimp Penaeus vannamei. Gene. 2019;706: 19–31. doi: 10.1016/j.gene.2019.04.066 31028869

[41] D Pankaj, K Tushna, G Samir, S Seema, R Krishanu. Atypical septate junctions maintain the somatic enclosure around maturing spermatids and prevent premature sperm release in Drosophila testis. Biol Open. 2019;8: 1–23. doi: 10.1242/bio.036939 30635267 PMC6398457

[42] Banerjee S, Sousa AD, Bhat MA. Organization and function of septate junctions: An evolutionary perspective. Cell Biochem Biophys. 2006;46: 65–77. doi: 10.1385/CBB:46:1:65 16943624

[43] Shinoharaa A, Shinohara M. Roles of RecA homologues Rad51 and Dmc1 during meiotic recombination. Cytogenet Genome Res. 2004;107: 201–207. doi: 10.1159/000080598 15467365

[44] Wongsurawat T, Leelatanawit R, Thamniemdee N, Uawisetwathana U, Karoonuthaisiri N, Menasveta P, et al. Identification of testis-relevant genes using in silico analysis from testis ESTs and cDNA microarray in the black tiger shrimp (*Penaeus monodon*). BMC Mol Biol. 2010;11: 1–15. doi: 10.1186/1471-2199-11-55 20696033 PMC2928233

[45] Okutsu T, Kang BJ, Miwa M, Yoshizaki G, Maeno Y, Wilder MN. Molecular cloning and characterization of Dmc1, a gene involved in gametogenesis, from the whiteleg shrimp *Litopenaeus vannamei*. Fish Sci. 2010;76: 961–969. doi: 10.1007/s12562-010-0295-6

[46] Inui T, Ohkubo T, Urade Y, Hayaishi O. Enhancement of lipocalin-type prostaglandin D synthase enzyme activity by guanidine hydrochloride. Biochem Biophys Res Commun. 1999;266: 641–646. doi: 10.1006/bbrc.1999.1881 10603301

[47] Helliwell RJA, Adams LF, Mitchell MD. Prostaglandin synthases: Recent developments and a novel hypothesis. Prostaglandins Leukot Essent Fat Acids. 2004;70: 101–113. doi: 10.1016/j.plefa.2003.04.002 14683687

[48] Wilhelm D, Hiramatsu R, Mizusaki H, Widjaja L, Combes AN, Kanai Y, et al. SOX9 regulates prostaglandin D synthase gene transcription in vivo to ensure testis development. J Biol Chem. 2007;282: 10553–10560. doi: 10.1074/jbc.M609578200 17277314

[49] Fang DA, Yang QZ, Duan JR, Wang Q, Zhang MY, Zhou YF, et al. Characteristic of PGDS potential regulation role on spermatogenesis in the Chinese mitten crab *Eriocheir sinensis*. Gene. 2014;543: 244–252. doi: 10.1016/j.gene.2014.04.010 24709109

[50] Kota J, Handy CR, Haidet AM, Montgomery CL, Eagle A, Rodino-Klapac LR, et al. Follistatin gene delivery enhances muscle growth and strength in nonhuman primates. Sci Transl Med. 2009;1: 1–8. doi: 10.1126/scitranslmed.3000112 20368179 PMC2852878

[51] Loongyai W, Avarre JC, Cerutti M, Lubzens E, Chotigeat W. Isolation and functional characterization of a new shrimp ovarian peritrophin with antimicrobial activity from *Fenneropenaeus merguiensis*. Mar Biotechnol. 2007;9: 624–637. doi: 10.1007/s10126-007-9019-z 17641929

[52] Spaziani EP, Hinsch GW, Edwards SC. Changes in prostaglandin E2 and F2α during vitellogenesis in the florida crayfish *Procambarus paeninsulanus*. J Comp Physiol B. 1993;163: 541–545. doi: 10.1007/BF00302112 8151012

[53] Spaziani EP, Hinsch GW. Variation in selected unsaturated fatty acids during vitellogenesis in the Florida freshwater crayfish *Procambarus paeninsulanus*. Invertebr Reprod Dev. 2011;32: 21–25. doi: 10.1080/07924259.1997.9672600

[54] Lim W, Song G. Differential expression of vitelline membrane outer layer protein 1: hormonal regulation of expression in the oviduct and in ovarian carcinomas from laying hens. Mol Cell Endocrinol. 2015;399: 250–258. doi: 10.1016/j.mce.2014.10.015 25458700

[55] Chen X, Yang H, Ruan Y, Zhou M, Liu J, Li Z, et al. Pacific white shrimp (*Litopenaeus vannamei*) vitelline membrane outer layer protein 1 (VMO1) is produced in the hepatopancreas and transported into ovarian oocytes during vitellogenesis. Gene. 2023;851: 147027. doi: 10.1016/J.GENE.2022.147027 36332838

[56] Ruan Y, Wong NK, Zhang X, Zhu C, Wu X, Ren C, et al. Vitellogenin receptor (VgR) mediates oocyte maturation and ovarian development in the Pacific white shrimp (*Litopenaeus vannamei*). Front Physiol. 2020;11: 485. doi: 10.3389/fphys.2020.00485 32499719 PMC7243368

[57] Ye YX, Pan PL, Xu JY, Shen ZF, Kang D, Lu JB, et al. Forkhead box transcription factor L2 activates Fcp3C to regulate insect chorion formation. Open Biol. 2017;7. doi: 10.1098/rsob.170061 28615473 PMC5493777

[58] Ayub Z, Ahmed M. A description of the ovarian development stages of penaeid shrimps from the coast of Pakistan. Aquac Res. 2002;33: 767–776. doi: 10.1046/j.1365-2109.2002.00715.x

[59] Peixoto S, Cavalli RO, D’Incao F, Milach ÂM, Wasielesky W. Ovarian maturation of wild *Farfantepenaeus paulensis* in relation to histological and visual changes. Aquac Res. 2003;34: 1255–1260. doi: 10.1046/j.1365-2109.2003.00933.x

[60] Wonglapsuwan M, Miyazaki T, Loongyai W, Chotigeat W. Characterization and biological activity of the ribosomal protein L10a of the white shrimp: F*enneropenaeus merguiensis* De Man during vitellogenesis. Mar Biotechnol. 2010;12: 230–240. doi: 10.1007/s10126-009-9220-3 19697087

[61] Makkapan W, Maikaeo L, Miyazaki T, Chotigeat W. Molecular mechanism of serotonin via methyl farnesoate in ovarian development of white shrimp: *Fenneropenaeus merguiensis* de Man. Aquaculture. 2011;321: 101–107. doi: 10.1016/j.aquaculture.2011.08.016

[62] Garza-Torres R, Campos-Ramos R, Maeda-Martínez AM. Organogenesis and subsequent development of the genital organs in female and male Pacific white shrimp *Penaeus* (*Litopenaeus*) *vannamei*. Aquaculture. 2009;296: 136–142. doi: 10.1016/j.aquaculture.2009.08.012

[63] K Yi K, Tsutsui N, Kawazoe I, Okumura T, Kaneko T, Aida K. Localization and developmental expression of mRNA for cortical rod protein in kuruma prawn *Marsupenaeus japonicus*. Zoolog Sci. 2005;22: 675–680. doi: 10.2108/zsj.22.675 15988163

[64] Quackenbush LS, Yolk synthesis in the marine shrimp, *Penaeus vannamei*. Comp Biochem Physiol Part A Physiol. 1992;103: 711–714. doi: 10.1016/0300-9629(92)90170-U

[65] Browdy CL, Fainzilber M, Tom M, Loya Y, Lubzens E. Vitellin synthesis in relation to oogenesis in in vitro‐incubated ovaries of *Penaeus semisulcatus* (crustacea, decapoda, penaeidae). J Exp Zool. 1990;255: 205–215. doi: 10.1002/jez.1402550209

[66] Tiu SHK, Hui JHL, Mak ASC, He JG, Chan SM. Equal contribution of hepatopancreas and ovary to the production of vitellogenin (PmVg1) transcripts in the tiger shrimp, *Penaeus monodon*. Aquaculture. 2006;254: 666–674. doi: 10.1016/j.aquaculture.2005.11.001

[67] Wonglapsuwan M, Phongdara A, Chotigeat W. Dynamic changes in gene expression during vitellogenic stages of the white shrimp: *Fenneropenaeus merguiensis* de Man. Aquac Res. 2009;40: 633–643. doi: 10.1111/j.1365-2109.2008.02136.x

[68] Tsukimura B. Crustacean vitellogenesis: its role in oocyte development1. Am Zool. 2001;41: 465–476. doi: 10.1668/0003-1569(2001)041[0465:CVIRIO]2.0.CO;2

[69] Garza-Torres R, Maeda-Martínez AM, Guerrero-Tortolero DA, Obregón-Barboza H, Campos-Ramos R. Description of meiosis in female and male pacific white shrimp *Litopenaeus vannamei* (Decapoda: Penaeidae). J Crustac Biol. 2011;31: 75–81. doi: 10.1651/10-3316.1

[70] Sagi A, Karp L, Milner Y, Cohen D, Kuris AM, Chang ES. Testicular thymidine incorporation in the prawn *Macrobrachium rosenbergii*: Molt cycle variation and ecdysteroid effects in vitro. J Exp Zool. 1991;259: 229–237. doi: 10.1002/jez.1402590212

[71] Sagi A, Milner Y, Cohen D. Spermatogenesis and Sperm Storage in the Testes of the Behaviorally Distinctive Male Morphotypes of *Macrobrachium rosenbergii* (Decapoda, Palaemonidae). Biol Bull. 1988;174: 330–336. doi: 10.2307/1541958

[72] Heitzmann JC, Diter A, AQUACOP. Spermatophore formation in the white shrimp, *Penaeus vannamei* Boone 1931: dependence on the intermoult cycle. Aquaculture. 1993;116: 91–98. doi: 10.1016/0044-8486(93)90225-N

[73] Jiang J, Yuan X, Qiu Q, Huang G, Jiang Q, Fu P, et al. Comparative transcriptome analysis of gonads for the identification of sex-related genes in giant freshwater prawns (*Macrobrachium rosenbergii*) using RNA sequencing. Genes (Basel). 2019;10: 1–18. doi: 10.3390/genes10121035 31835875 PMC6947849

[74] Yuan Y, Lin J, Tan X, Shi X, Fang S, Zhang Y, et al. Identification and sexually dimorphic expression of a FoxL2-like gene in Mud crab (*Scylla paramamosain*): potential roles in male differentiation and development. Thalassas: An International Journal of Marine Sciences. 2021;37: 131–140. doi: 10.1007/s41208-020-00249-1

