# Supplementary figures and images for: Transcriptome profiling of banana shrimp (Fenneropenaeus merguiensis) ovaries and testes: Insights into FoxL2
Source: PLoS One. 2023 Oct 12;18(10):e0292782. doi: 10.1371/journal.pone.0292782 (PMC10569530; doi:10.1371/journal.pone.0292782)

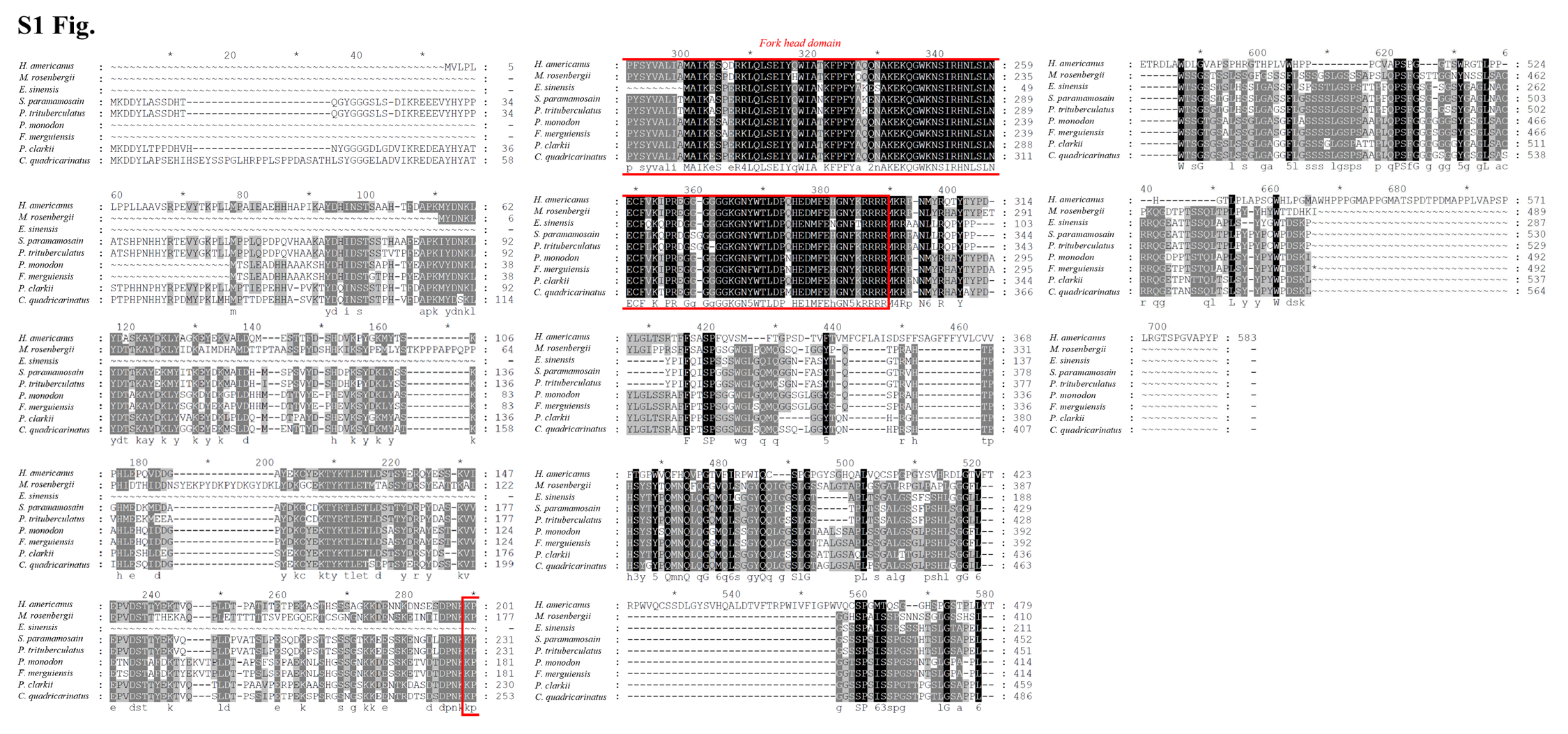

Supplement: S1 Fig — Multiple alignments of the deduced amino acid sequence of the FoxL2 gene, GenBank accession no. OQ870552, with H. americanus: accession no. KAG7154875.1, M. rosenbergii: accession no. USJ75257.1, E. sinensis: accession no. AIS92518.1, S. paramamosain: accession no. QQY98966.1, P. trituberculatus: accession no. WAA68168.1, P. monodon: accession no. XP_037795163.1, P. clarkii: accession no. ALD48735.1, and C. quadricarinatus: accession no. UWX37250.1. The red boxes represent the FoxL2 conserved domain. (TIF) [file pone.0292782.s001.tif]
